# Lifespan Extension by Methionine Restriction Requires Autophagy-Dependent Vacuolar Acidification

**DOI:** 10.1371/journal.pgen.1004347

**Published:** 2014-05-01

**Authors:** Christoph Ruckenstuhl, Christine Netzberger, Iryna Entfellner, Didac Carmona-Gutierrez, Thomas Kickenweiz, Slaven Stekovic, Christina Gleixner, Christian Schmid, Lisa Klug, Alice G. Sorgo, Tobias Eisenberg, Sabrina Büttner, Guillermo Mariño, Rafal Koziel, Pidder Jansen-Dürr, Kai-Uwe Fröhlich, Guido Kroemer, Frank Madeo

**Affiliations:** 1 Institute for Molecular Biosciences, University of Graz, Graz, Austria; 2 INSERM, U848, Villejuif, France; 3 Institut Gustave Roussy, Villejuif, France; 4 Université Paris Sud, Paris 11, Villejuif, France; 5 Institute for Biomedical Aging Research (IBA), Austrian Academy of Sciences, Innsbruck, Austria; 6 Metabolomics Platform, Institut Gustave Roussy, Villejuif, France; 7 Centre de Recherche des Cordeliers, Paris, France; 8 Pôle de Biologie, Hôpital Européen Georges Pompidou, AP-HP, Paris, France; 9 Université Paris Descartes, Paris 5, Paris, France; Stanford University Medical Center, United States of America

## Abstract

Reduced supply of the amino acid methionine increases longevity across species through an as yet elusive mechanism. Here, we report that methionine restriction (MetR) extends yeast chronological lifespan in an autophagy-dependent manner. Single deletion of several genes essential for autophagy (*ATG5*, *ATG7* or *ATG8*) fully abolished the longevity-enhancing capacity of MetR. While pharmacological or genetic inhibition of *TOR1* increased lifespan in methionine-prototroph yeast, *TOR1* suppression failed to extend the longevity of methionine-restricted yeast cells. Notably, vacuole-acidity was specifically enhanced by MetR, a phenotype that essentially required autophagy. Overexpression of vacuolar ATPase components (Vma1p or Vph2p) suffices to increase chronological lifespan of methionine-prototrophic yeast. In contrast, lifespan extension upon MetR was prevented by inhibition of vacuolar acidity upon disruption of the vacuolar ATPase. In conclusion, autophagy promotes lifespan extension upon MetR and requires the subsequent stimulation of vacuolar acidification, while it is epistatic to the equally autophagy-dependent anti-aging pathway triggered by *TOR1* inhibition or deletion.

## Author Summary

Health- or lifespan-prolonging regimes would be beneficial at both the individual and the social level. Nevertheless, up to date only very few experimental settings have been proven to promote longevity in mammals. Among them is the reduction of food intake (caloric restriction) or the pharmacological administration of caloric restriction mimetics like rapamycin. The latter one, however, is accompanied by not yet fully estimated and undesirable side effects. In contrast, the limitation of one specific amino acid, namely methionine, which has also been demonstrated to elongate the lifespan of mammals, has the advantage of being a well applicable regime. Therefore, understanding the underlying mechanism of the anti-aging effects of methionine restriction is of crucial importance. With the help of the model organism yeast, we show that limitation in methionine drastically enhances autophagy, a cellular process of self-digestion that is also switched on during caloric restriction. Moreover, we demonstrate that this occurs in causal conjunction with an efficient pH decrease in the organelle responsible for the digestive capacity of the cell (the vacuole). Finally, we prove that autophagy-dependent vacuolar acidification is necessary for methionine restriction-mediated lifespan extension.

## Introduction

Methionine restriction (MetR) has been long known to enhance lifespan in various organisms, including mammals [1], [2]. Nevertheless, the mechanisms underlying this phenomenon are poorly understood. Previous studies have mainly focused on MetR-induced alterations of the function and composition of respiratory chain complexes in mitochondria, although no clear cause-effect relationship between these effects and the beneficial impact on longevity could be established [3]–[5].

Given that MetR represents a regime that limits availability of an amino acid, we wondered if the resulting longevity effect might include the involvement of autophagy [6], which is known to play a crucial role in cells that are stressed by damage or limited nutrient supply [7]. Only recently, autophagy has been shown to play an important role for lifespan extension by treatment with spermidine, rapamycin, or resveratrol, as well as by depletion of the p53 ortholog from *Caenorhabditis elegans*, the inhibition of IGF signaling, and the overexpression of sirtuin [8]–[12]. The autophagic process depends on the vacuolar proteolytic activity, which is determined by vacuolar acidification [13], [14]. Based on these premises, we decided to analyze whether macroautophagy (hereafter referred to as autophagy) is also induced under conditions of MetR and if autophagy induction contributes to MetR-induced lifespan extension. In addition, we analyzed the extent of vacuolar acidification, which has been recently shown to be crucial for lifespan extension in replicatively aging cells [15]. To tackle these questions, we decided to use baker's yeast (*Saccharomyces cerevisiae*) as a model system, since (i) it constitutes a well-established (chronological) aging model [16]; (ii) autophagy was discovered and largely uncovered in this model [17]–[19]; and (iii) MetR can be reliably controlled by virtue of media supplementation and the deletion of genes involved in its biosynthesis [20].

Here, we report that MetR causes an increase in chronological lifespan (CLS) that depends on the enhanced vacuole acidification that follows autophagy stimulation.

## Results

### Methionine limitation causes longevity during yeast chronological aging

**Figure 1:**
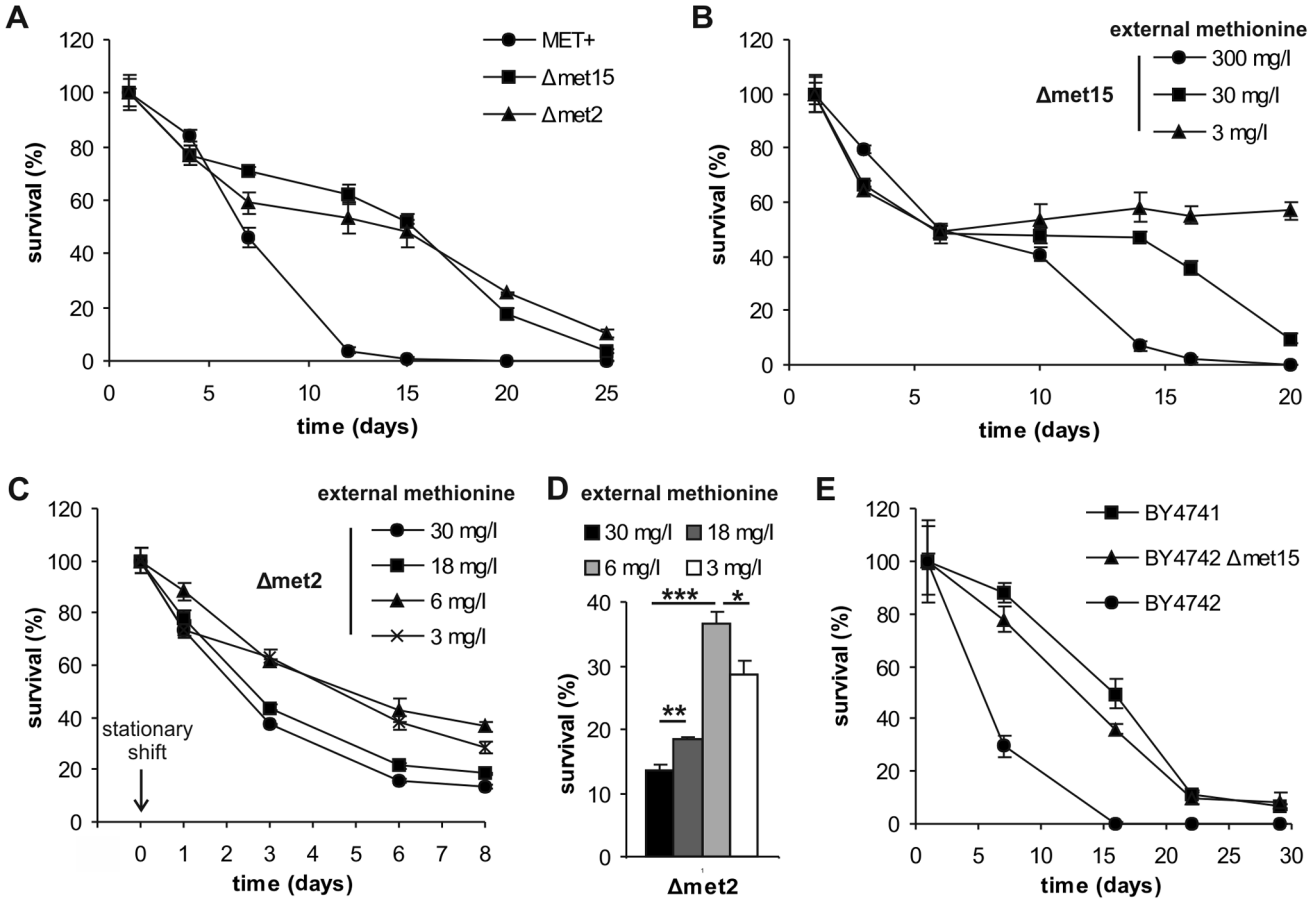
Methionine determines yeast chronological lifespan. (A) Chronological aging of methionine prototroph (MET^+^), semi-auxotroph (Δ*met15*) and auxotroph (Δ*met2*) isogenic yeast strains in SCD media supplemented with all amino acids (aa). Cell survival was estimated as colony formation of 500 cells plated at given time points, normalized to cell survival on day one (n = 4). (B) Chronological aging of Δ*met15* strain, in SCD media supplemented with all aa except for methionine which was added at given concentrations. Cell survival of 500 cells plated at given time points, normalized to cell survival on day one (n = 4). (C) *MET2* deletion strain (Δ*met2*) was grown to stationary phase in SCD (supplemented with all aa) and shifted to SCD media with different methionine concentrations. Cell survival of 500 cells plated at given time points, normalized to cell survival before the shift (n = 4). (D) Day 8 from experiment shown in C (n = 4). (E) Chronological aging of EUROSCARF BY4741 (also used above as Δ*met15* reference strain) and mating type α wild type strain BY4742, as well as a methionine semi-auxotrophic variant thereof (BY4742 Δ*met15*), in SCD media supplemented with all aa. Cell survival of 500 cells plated at given time points, normalized to cell survival on day one (n = 3). See also Figure S1.

To determine whether MetR influences yeast CLS, three different strains were used: (i) a MET^+^ strain that is fully competent in synthesizing methionine, (ii) a knockout strain deleted in *met15* that suffers only a moderate defect in methionine biosynthesis due to an intact salvage pathway mediated via O-acetyl-homoserine (the reaction product of Met2p), and (iii) a *met2* deletion strain fully devoid of a *de novo* methionine synthesis and hence strictly dependent on externally supplied methionine [20]. These three strains are otherwise isogenic (Table S1). The methionine-prototroph strain (MET^+^) exhibited a rather short CLS of about 10 to 15 days, while the two methionine-auxotroph strains with limited (Δ*met15*) and no (Δ*met2*) endogenous methionine biosynthesis displayed enhanced lifespans of up to 25 days (Figure 1A). Of note, these chronological aging experiments were performed in synthetic complete medium supplemented with 30 mg/l methionine. Using this medium, all three strains showed equivalent cell counts during aging experiments (Figure S1A) and cell cycle arrest in G0/G1 was comparable (Figure S1B), therefore excluding potential artifacts secondary to differential growth rates and nutrient consumption. Furthermore, chronological aging experiments of MET^+^, Δ*met15* and Δ*met2* revealed only marginal differences in external media pH (Figure S1C), thus showing that external pH effects do not play a major role in this scenario [21]. Accordingly, it has been recently shown that lifespan-enhancing conditions do not necessarily correlate with changes in pH [22].

We next determined the influence of external methionine availability on the MET^+^, Δ*met15*, and Δ*met2* strains by using media supplemented with varying methionine concentrations. Higher levels of supplemented methionine led to decreased survival in Δ*met15* and Δ*met2* strains (Figure 1B and Figure S1D) whereas a reduction of methionine led to improved longevity - especially of long-term survival - of the semi-auxotrophic strain (Δ*met15*, Figure 1B, cell-counts Figure S1E), but not of the prototrophic strain (MET^+^) (Figure S1F, cell-counts Figure S1G). Of note, lower amounts of cysteine (a downstream product of methionine biosynthesis) did not increase CLS of Δ*met15* whereas high amounts shorten CLS, potentially via formation of methionine by transsulfuration (Figure S1H) [20]. Also note that reduced methionine levels were not used in the case of the auxotrophic Δ*met2* strain because cell counts, grown in the presence of 3 mg/l, of this strain are ten times lower compared to 30 mg/l standard conditions (Figure S1I). Thus, to verify effects of low levels of methionine on the fully auxotrophic Δ*met2* strain during chronological aging, cells were grown to stationary phase (24 hours) in media with excess methionine (30 mg/l) to support normal growth, and then transferred to media with lower methionine concentrations (Figure 1C and D). Δ*met2* cell cultures transferred to media with high methionine concentrations exhibited accelerated aging, while lowering methionine concentrations increased longevity. Optimal cell survival was reached with around 6 mg/l externally supplied methionine (Figure 1C and D). Thus MetR during chronological aging can only be achieved by combining both, the deletion of specific genes involved in its biosynthesis and external methionine supplementation. Moreover, MetR resulted in reduced phosphatidylserine externalization (a typical sign of apoptosis) and improved plasma membrane integrity (which is disrupted in necrosis), as determined by AnnexinV/PI-costaining (Figure S2A). The levels of reactive oxygen species (ROS), which are suspected mediators of cellular aging, were determined by monitoring the conversion of dihydroethidium (DHE) to fluorescent ethidium (Eth), as driven by superoxide anion radicals. ROS levels were clearly diminished in Δ*met15* and Δ*met2* strains (Figure S2B).

Intriguingly, one genetic difference (beside the functionality of their *LYS2* gene-product and a different mating type) of the frequently used wild type strains of the EUROSCARF strain collection, BY4741 and BY4742, also affects their ability to produce methionine. The long-lived BY4741 strain harbors a deletion of *MET15* (and was also used in the above described experiments) whereas the short-lived BY4742 strain is methionine-prototroph. Deletion of *MET15* from BY4742 reestablished a long-lived phenotype in chronological aging experiments, indicating that the short life expectancy of this strain is indeed due to its ability to synthesize methionine (Figure 1E).

We conclude that the amount of methionine availability – as determined by *de novo* synthesis or external supply – dramatically influences the survival of chronologically aging yeast and protects against apoptosis/necrosis.

### Autophagy is specifically induced upon methionine restriction

**Figure 2:**
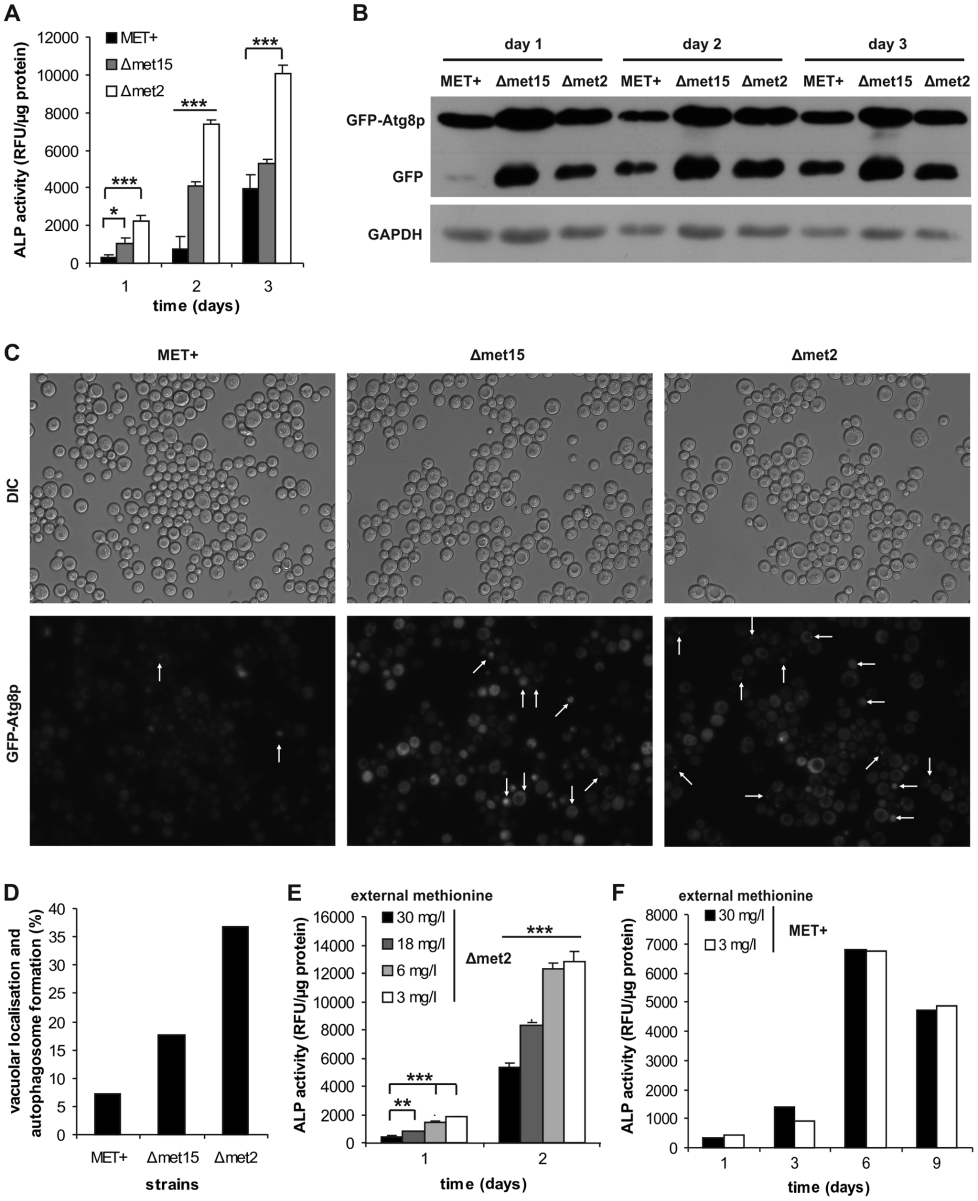
MetR specifically regulates induction of autophagy. MET^+^, Δ*met15* and Δ*met2* strains from chronological aging experiments were analyzed for vacuolar ALP activity (with a fluorescent plate reader) (A) (n = 6), and GFP-Atg8p processing (by Western-blot analysis) (B). (C) GFP-Atg8p localization was determined by using fluorescent microscopy (white arrows indicate vacuolar localization or autophagosome formation) and statistical analysis thereof (330–600 cells of each GFP-Atg8p expressing strain were evaluated from two independent samples) (D). (E) *MET2* deletion strain (Δ*met2*) was grown to stationary phase in SCD (supplemented with all aa) and shifted to SCD media with given methionine concentrations. Autophagy was measured by means of ALP activity with a fluorescent plate reader (Tecan, Genios Pro) (n = 6). (F) ALP assays of chronological aging of MET^+^ strain, in SCD media supplemented with all aa except for methionine which was added at given concentrations (n = 2). See also Figure S3.

Since autophagy might be one of the major pathways responsible for lifespan extension in various organisms and under diverse circumstances [10], [23] we determined the rate of autophagy within the cell cultures. During the first days of chronological aging, alkaline phosphatase (ALP) activity (which measures the activity of cytosolic ALP which is delivered to the vacuole exclusively via autophagy) was significantly higher in Δ*met15* and Δ*met2* strains compared to the MET^+^ strain (Figure 2A). Accordingly, vacuolar processing of GFP-tagged Atg8p, which takes place under autophagic conditions for recycling of the protein, was elevated upon MetR, as visualized by immunoblotting (Figure 2B). Furthermore, the localization of GFP-tagged Atg8p, a protein essential for the autophagic process, which is normally evenly distributed in the cytoplasm (see MET^+^ strain), showed punctuate and/or vacuolar localization in Δ*met15* and Δ*met2* strains during chronological aging (shown for day 2 in Figure 2C and D). This reflects enhanced autophagosome formation and Atg8p-delivery to the vacuole. Of note, differences in the number of puncta per cell compared to the number of positive vacuoles per cell possibly reflect dynamics of this process and are subject to changes over time. To exclude effects of distinct Atg8p levels deriving from possible differences in synthesis capabilities of the different strain backgrounds, we performed overexpression studies of Atg8p in MET^+^. No differences in CLS were observed (Figure S3A). Next, we determined the autophagic flux (ALP activity) under different levels of MetR by transferring stationary Δ*met2* cell cultures grown in excess of methionine to media supplemented with varying methionine concentrations that showed beneficial effects on longevity (see above and Figure 1C and D). A clear inverse correlation of methionine concentration and autophagy level was visible, reaching a nearly ten-fold difference in early onset of autophagy between a high methionine concentration (30 mg/l) and cells exposed to low concentrations (3 to 6 mg/l) of methionine (Figure 2E). In addition, the MET^+^ strain showed no altered ALP activity when grown in the presence of 3 mg/l methionine (Figure 2F), as opposed to the *met15* deletion strain where ALP activity was strongly up-regulated (Figure S3B). Accordingly, the Δ*met2* strain grown in the presence of high levels of external methionine showed decreased autophagy (Figure S3C). We conclude that methionine restriction specifically enhances autophagy during yeast chronological aging.

### Autophagy is essential for methionine restriction-induced longevity

**Figure 3:**
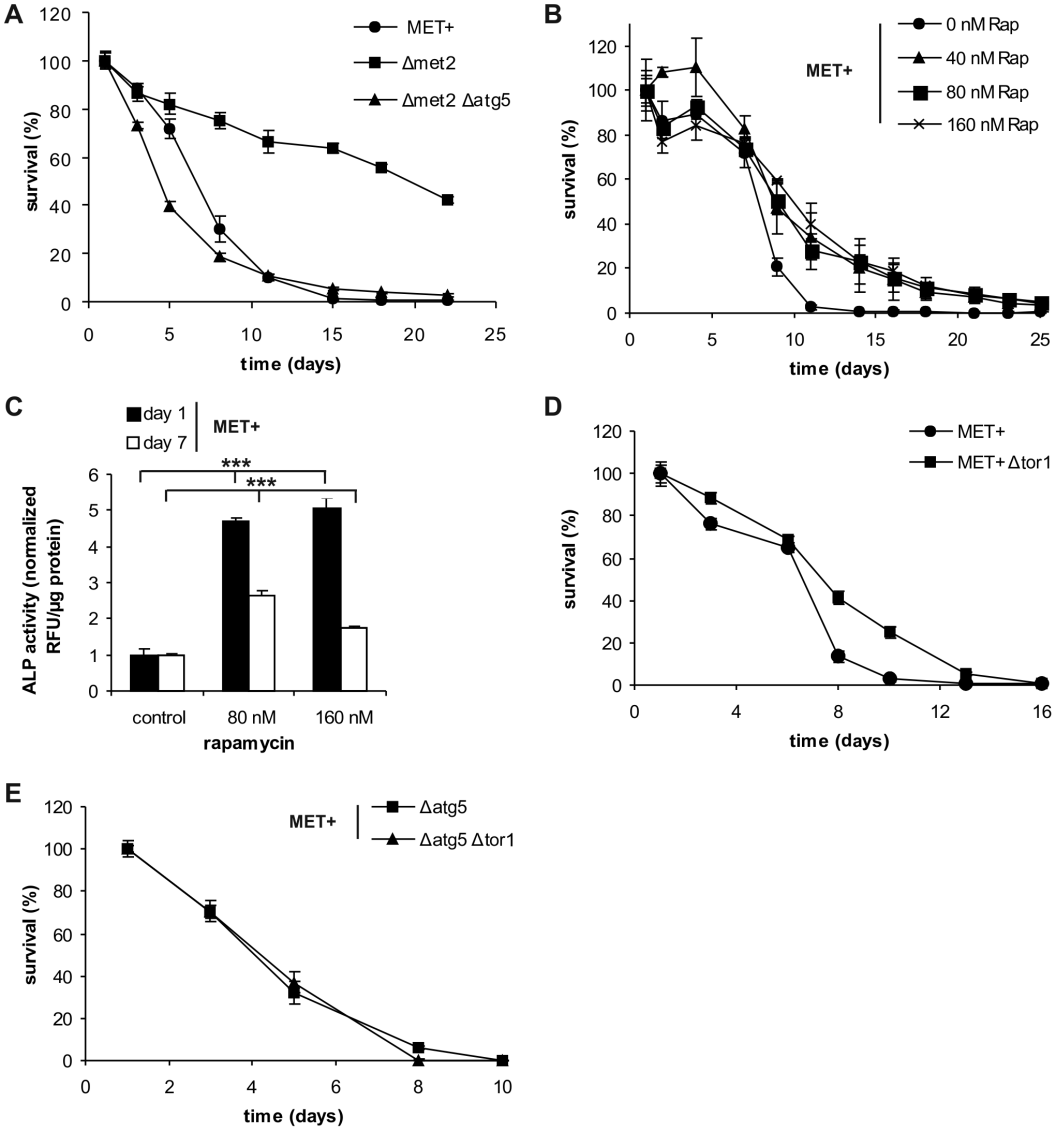
Autophagy is crucial for MetR-mediated longevity. (A) Chronological aging of *MET2* deletion strains carrying an additional gene deletion (Δ*atg5*) and MET^+^ strain in SCD media supplemented with all aa. Cell survival of 500 cells plated at given time points, normalized to cell survival on day one (n = 6). (B) Chronological aging of MET^+^ strain treated with indicated amounts of rapamycin (Rap). Cell survival of 500 cells plated at given time points, normalized to cell survival on day one (n = 3; p***). Autophagy was measured by means of ALP activity with a fluorescent plate reader (Tecan, Genios Pro) and normalized to untreated controls at indicated time points (also compare to Figure 4E) (C) (n = 4). (D) Chronological aging of MET^+^ strain deleted for *TOR1*. Cell survival of 500 cells plated at given time points, normalized to cell survival on day one (n = 6; p***). (E) Chronological aging of the MET^+^ strain deleted for *TOR1* and *ATG5* or *ATG5* alone. Cell survival of 500 cells plated at given time points, normalized to cell survival on day one (n = 4–6). See also Figure S4.

To verify whether autophagy truly impacts rather than only correlating with lifespan extension upon MetR, we subjected several strains harboring single deletions of genes necessary for MetR-triggered autophagy. Deletion of *ATG5*, *ATG7* or *ATG8*, abolished the gain in longevity that was normally conferred by MetR. In both, the Δ*met15* and the Δ*met2* strains, lifespan was drastically shortened upon ATG gene deletions reaching comparable or even lower survival levels than those of the corresponding MET^+^ strain (Figure 3A and S4A, B). Of note, an additional *RAS2* deletion, a well-established longevity-mediating mutation, in an autophagy-deficient Δ*met2* strain (Δ*met2* Δ*atg5*) led to increased longevity, clearly indicating that autophagy-deficient strains are not *per se* unable to survive longer but that other (non-autophagic) pro-survival mechanisms are functional (Fig. S4C). Importantly, survival of the MET^+^ strain deleted for *ATG5*, *ATG7* or *ATG8*, did not alter CLS during the first three days. Only after day 3, when autophagy started to increase (Figure 2A and B) and thus seemed to become a physiological need, CLS was shortened (Figure S4D).

Next, we determined whether rapamycin, an established pharmacological inhibitor of TOR and inducer of autophagy, could enhance lifespan of the MET^+^ strain. Rapamycin treatment indeed extended the longevity of the MET^+^ strain (Figure 3B). In accordance, the autophagy rate under these conditions was strongly induced (2 to 5 times) as measured via ALP activity (Figure 3C). To minimize its effects on growth behavior, rapamycin was added during mid-log phase (8 hours after inoculation of the main culture). Therefore, the positive effect on chronological survival is neither mediated by possible growth-delays (Figure S4E) [8] nor by an enhancement of respiration in the logarithmic growth phase (Figure S4F) [24]. Similar to the pharmacologically mediated inhibition of the TOR pathway (by rapamycin), genetic ablation of *TOR1*, the initiator-kinase of the autophagy-repressive TOR pathway, resulted in increased CLS of MET^+^ cells (Figure 3D). The additional deletion of ATG genes essential for autophagy (*ATG5*, *ATG7*, or *ATG8*) prevented the positive effects of *TOR1* deletion in the methionine prototrophic strain (Figure 3E and Figure S4G and H), as was already shown for rapamycin mediated lonegvity [8]. We conclude that autophagy is crucial for MetR-induced longevity.

### MetR-induced autophagy is epistatic to TOR inhibition

**Figure 4:**
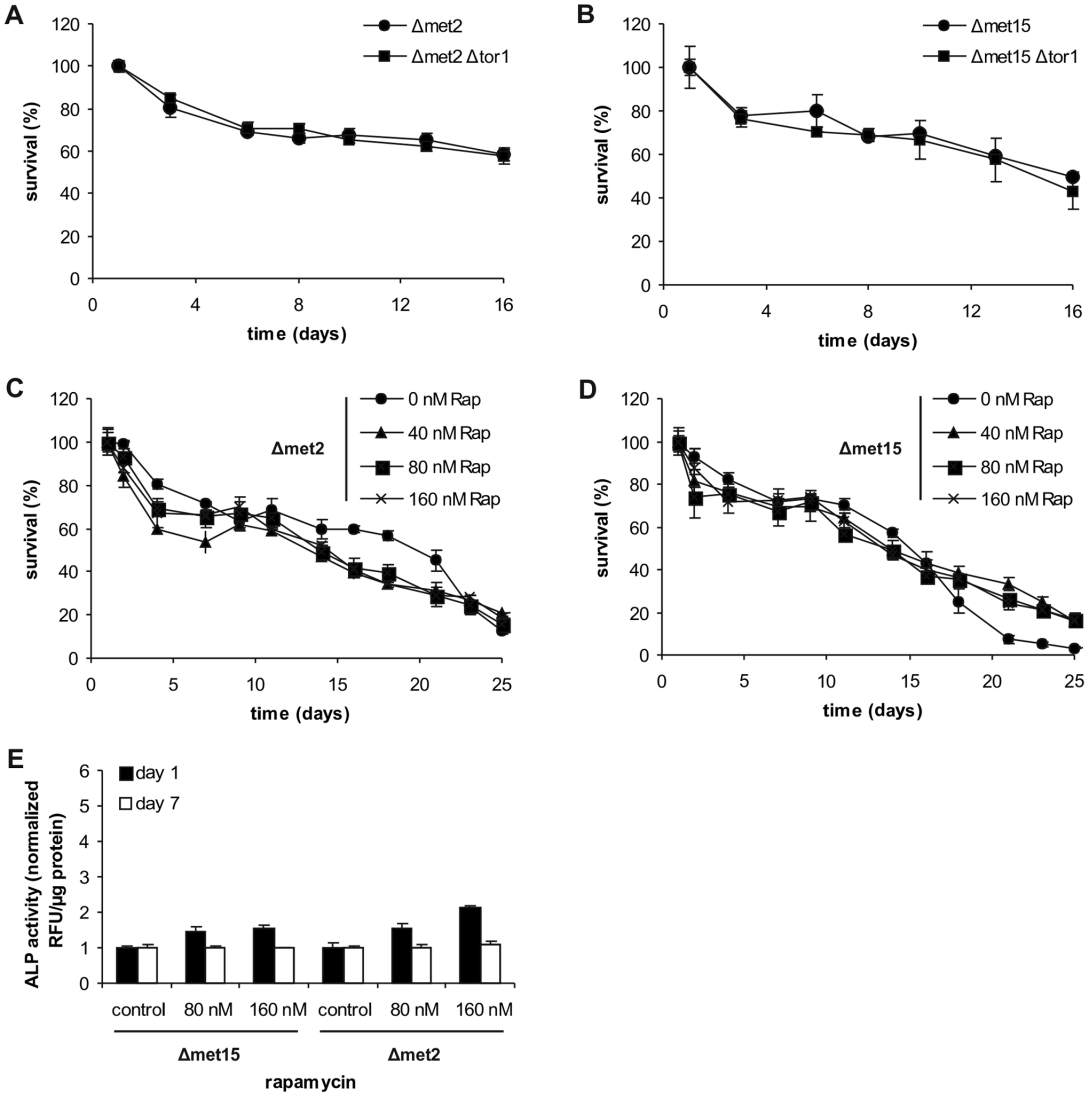
MetR is epistatic to other longevity treatments involving *TOR1* inhibition. (A and B) Chronological aging of *MET2* and *MET15* deletion strains deleted for *TOR1*. Cell survival of 500 cells plated at given time points, normalized to cell survival on day one (n = 6). Chronological aging of *MET2* (C) and *MET15* (D) deletion strains treated with indicated amounts of rapamycin (Rap). Cell survival of 500 cells plated at given time points, normalized to cell survival on day one (n = 3). Autophagy was measured by means of ALP activity with a fluorescent plate reader (Tecan, Genios Pro) and normalized to untreated controls at indicated time points (also compare to Figure 3C) (E) (n = 4). See also Figure S5.

Because the TOR pathway is one of the major sensors for (external) amino acid availability, we asked whether lifespan extension under MetR conditions could be further enhanced by TOR inhibition. For this purpose, we deleted *TOR1* in both the methionine-auxotrophic (Δ*met2*) and the semi-auxotrophic strains (Δ*met15*). Genetic ablation of the TOR pathway had no positive influence on survival (Figure 4A and B). This irresponsiveness seems to be independent from ROS generation since addition of low doses of glutathione did not affect CLS of Δ*met2* and Δ*met15* strains (Figure S5A and B). Intriguingly, external starvation for methionine in the Δ*met2*Δ*tor1* strain led to a small increase of autophagy on day 1, which became more pronounced with ongoing age (Figure S5C). This possibly shows that initial autophagy induction upon MetR is strongly dependent on *TOR1* whereas its maintenance might be additionally supported by *TOR1-*independent mechanism(s). Pharmacological inhibition of the TOR pathway (via rapamycin) under the very same conditions as described for the MET^+^ strain also failed to increase longevity of the Δ*met2* strain and had only rather small positive effects on the Δ*met15* strain (Figure 4C and D). Of note, a stronger increase in cell count after day 1 was observed for all strains treated with rapamycin, irrespective of its impact on CLS (Figure S4E and S5D, E). Moreover, rapamycin treatment only marginally increased autophagy rates at day 1, and had no effects at day 7 (measured by means of ALP activity) during Δ*met15* and Δ*met2* chronological aging (Figure 4E). This was presumably the case because autophagy was already strongly stimulated. In contrast, rapamycin enhanced autophagy rates in the methionine-prototroph strain (MET^+^) to factors of up to 5, on day 1, and 2 to 3, on day 7 (see above and compare Figure 3C and 4E). We conclude that TOR inhibition and MetR are, at least partly, part of the same anti-aging pathway.

### MetR increases the number of cells with acidic vacuoles in an autophagy-dependent manner

**Figure 5:**
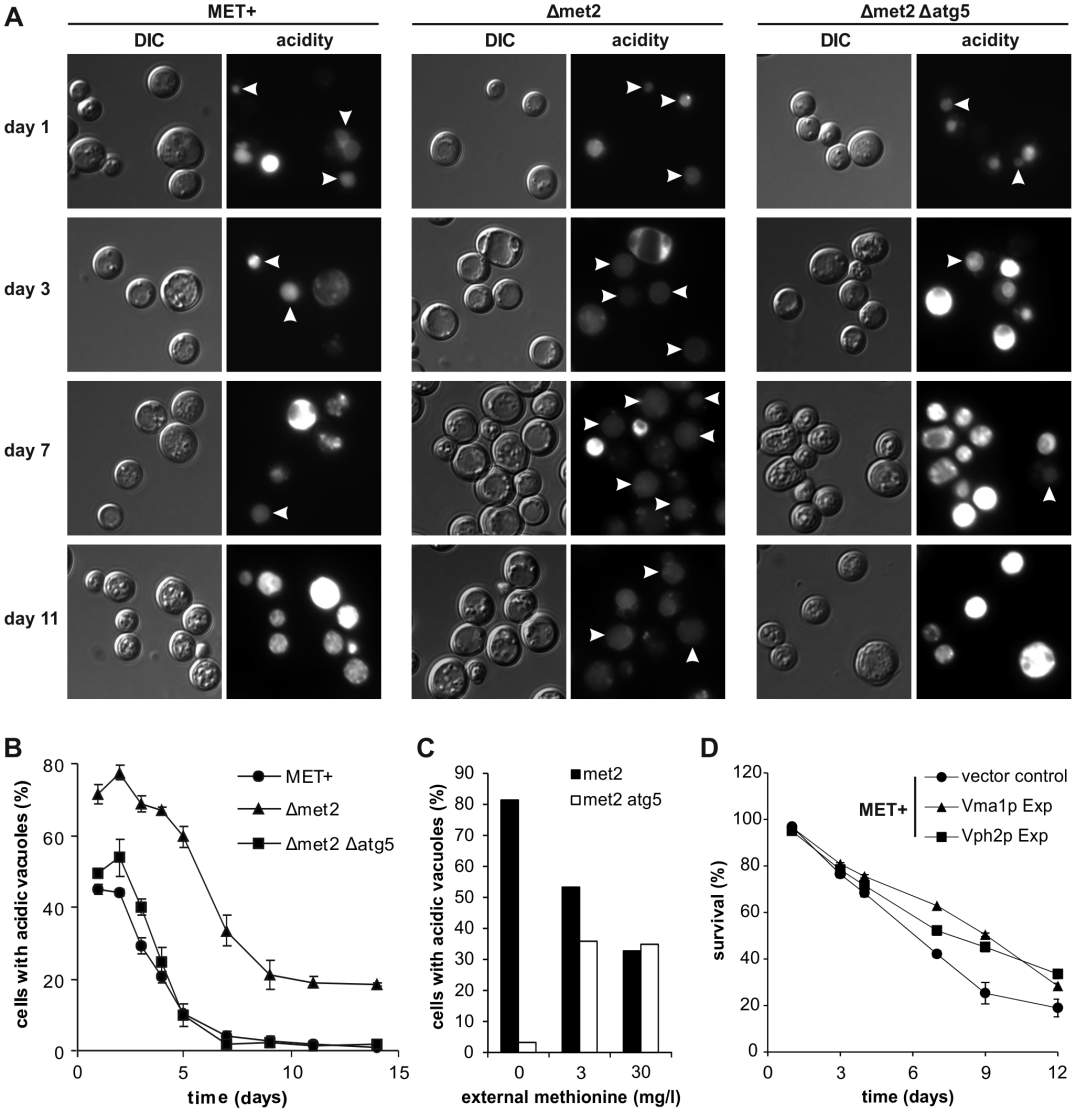
MetR enhancement of vacuolar acidification is autophagy-dependent and necessary for longevity. Fluorescent microscopy of acidic vacuoles during chronological aging of MET+, Δ*met2*, and Δ*met2*/Δ*atg5* strains, by means of quinacrine accumulation and statistical analysis thereof. (>1000 cells of each strain from 3 to 5 independent samples at each time point were evaluated. Only cells with acidic vacuoles without an additionally stained cytoplasm were counted as positive, resulting in cell counts that represent cells which have a clearly intact pH-homeostasis. Positively counted cells are indicated by white arrowheads) (A and B). (C) Statistical analysis of fluorescent microscopy of acidic vacuoles by means of quinacrine accumulation. Strains Δ*met2* and Δ*met2*/Δ*atg5* were grown to stationary phase under excess of methionine and shifted to media with the indicated amounts of methionine (>500 cells from each strain from 2 independent samples) and assayed for quinacrine accumulation after ∼20 hours (D) Chronological aging of the MET^+^ strain overexpressing Vma1p or Vph2p. Cell death was measured via propidium iodide staining of cells that have lost integrity and subsequent flow cytometry analysis (BD LSRFortessa) (n = 6 to 8). See also Figure S6.

The downstream target of autophagy is the vacuole. Thus, we mused if MetR-induced autophagy could enhance the degree of acidic vacuoles within the cell population. For this purpose, we stained chronologically aged MET^+^ or Δ*met2* cells with quinacrine, the most widely used and highly specific stain for acidic cell compartments and counted for cells where only the vacuole was stained. The Δ*met2* strain showed a 20% increase in cells harboring acidic vacuoles during chronological aging compared to the MET^+^ strain (Figure 5A and B). This increase was autophagy-dependent since it was completely abolished in a Δ*met2* strain lacking *ATG5* and thus autophagy-deficient (Figure 5A and B). Of note, cells showing a very bright quinacrine staining (older cells from MET^+^ and Δ*met2/*Δ*atg5*, Figure 5A) represent cells with acidic cytoplasm, which harbor almost no intact vacuoles as demonstrated with quinacrine-stained cells expressing a chromosomal mCherry-tagged version of the vacuolar membrane-located Vph1p (Figure S6A). To further show a direct regulation of vacuolar acidity upon MetR, through autophagy, we shifted Δ*met2* or Δ*met2*/Δ*atg5* cells to media with different methionine concentrations. The amount of cells with only acidic vacuoles was strictly dependent on the amount of supplemented methionine: ∼80% when shifted to media lacking methionine, ∼55% on 3 mg/l, and ∼35% on 30 mg/l methionine (Figure 5C). This dependency was blocked by an additional *ATG5* deletion, which causally links vacuolar acidification to MetR-induced autophagy (Figure 5C). Moreover, the MET^+^ strain grown in the presence of rapamycin, showed an increased proportion of cells harboring acidic vacuoles during aging (Figure S6B), in line with the beneficial effects of this pharmacological intervention on survival and increased autophagy (Figure 3B and C). Of note, starting at day 3 we could observe that the MET^+^ strain showed distinctly more vacuolar cargo compared to Δ*met2*, which is probably due to limited clearance. We conclude that autophagy is sufficient to significantly enhance the proportion of cells harboring acidic vacuoles during yeast chronological aging.

### Blockage of vacuole acidification largely ameliorates positive effects of MetR on longevity while overexpression of v-ATPase components increases longevity

**Figure 6:**
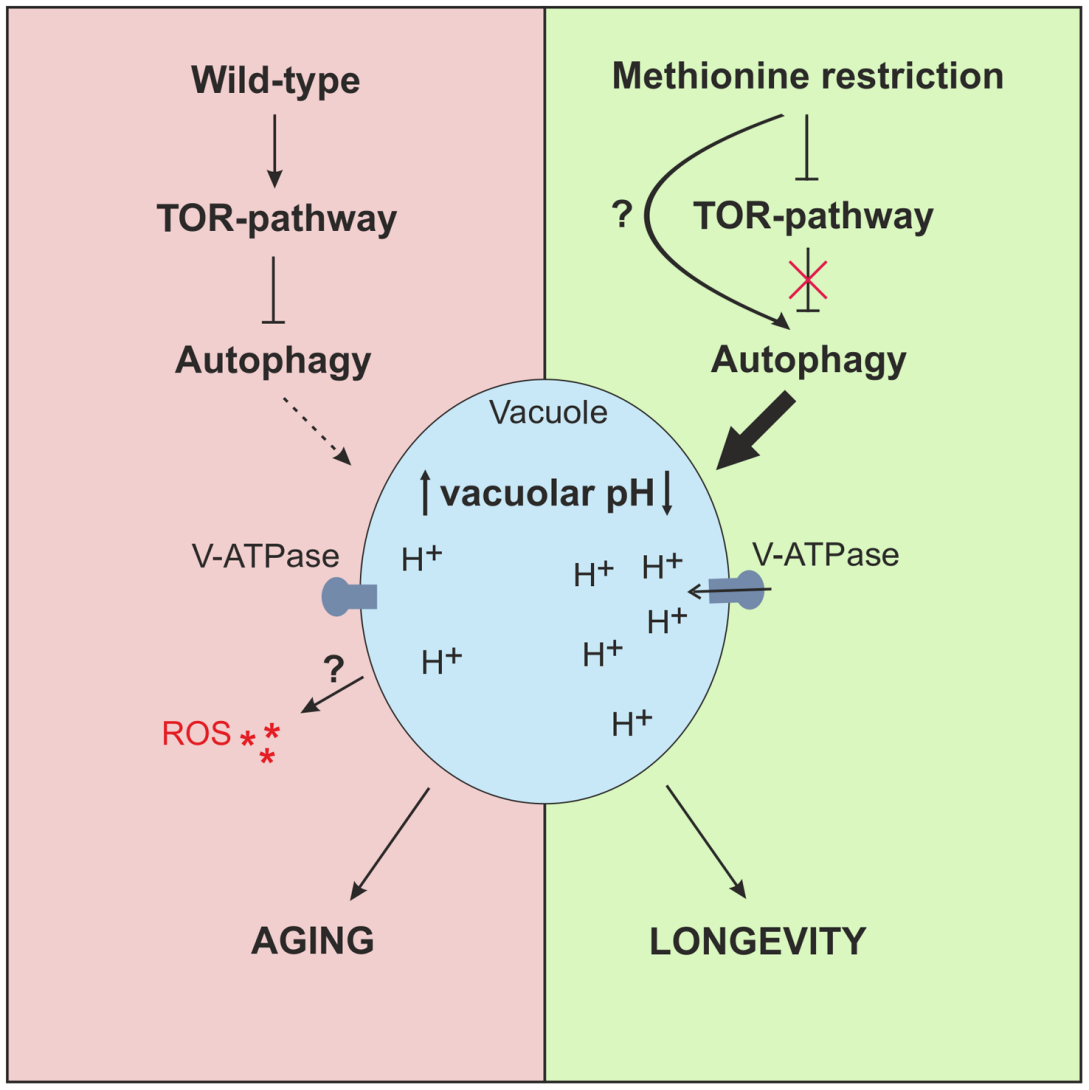
Model of MetR-mediated longevity. MetR specifically enhances autophagy, either by interfering upstream of TOR-pathway or presumably by impinging on (metabolic) pathways that potentially target autophagy directly, downstream of the TOR-pathway. MetR-specific vacuolar acidification is dependent on autophagy and elongates CLS. High levels of methionine inhibit autophagy induction during early phases of chronological aging, enhancing ROS and diminishing acidic vacuoles in a cell population, which leads to cell death.

Interestingly, an enhanced proportion of cells bearing an acidic vacuole has been recently shown to be crucial for improved replicative longevity by overexpressing *VMA1* or *VPH2* and thus increasing vacuolar acidity [15]. To explore whether such causal connection is also present between the enhancement of acidic vacuoles and the extended lifespan by MetR-induced autophagy, we overexpressed Vma1p, a part of the vacuolar ATPase (v-ATPase) or Vph2p, essential for v-ATPase assembly [25]–[27] in the MET^+^ strain. Overexpression of both proteins led to increased CLS (Figure 5D). Moreover a Δ*met2* strain deleted for *VPH2* showed diminished survival during chronological aging resembling more closely the MET^+^ strain (Figure S6C). It should be noted that a deletion leading to no detectable acidic vacuoles via quinacrine staining (Figure S6D), causes pleiotropic effects [28] and thus results must be interpreted cautiously. Still and supporting our knockout results, overexpression of Vph2p or Vma1p in the methionine-auxotrophic Δ*met2* strain did not improve CLS (Figure S6E). These results support a model, in which MetR-induced autophagy regulates vacuolar acidity which in turn promotes longevity (Figure 6). This places MetR-induced autophagy at the hub of vacuolar acidification and its positive effects on chronological survival, which are at least partly responsible for the positive effects observed on longevity through MetR (starvation for methionine).

## Discussion

Using *S. cerevisiae*, the key model organism in which autophagy was first functionally described and genetically dissected [17]–[19], we demonstrate that methionine restriction (MetR), unlike restriction in other amino acids such as leucine [6], [29], promotes clonogenic survival during chronological aging. MetR inhibits the ROS overproduction, as well as the aging-associated mortality by both apoptosis and necrosis. MetR shares analogies to limitations in elemental nutrients such as phosphor and sulfate, which induce a specific cell cycle arrest [30]. However, we could not find any signs of cell cycle blockade and our experiments were performed under conditions that fully supported growth to stationary phase in both methionine auxotrophic (Δ*met2*) and semi-auxotrophic (Δ*met15*) strains. Although there were no discernible cell cycle effects, MetR-induced lifespan extension correlated with enhanced autophagy, and the positive effect of MetR on longevity was lost when essential ATG genes were deleted. Accordingly, pharmacological or genetic inhibition of the TOR-pathway (and thus autophagy induction) enhanced CLS of a methionine-prototroph strain (MET^+^) but failed to do so in the methionine-auxotroph strains Δ*met2* and Δ*met15*. This epistatic analysis fully validates the concept that the beneficial effects of MetR on longevity are mediated by autophagy.

Only recently, two new mechanisms for autophagy regulation were described: (i) a methionine-related one, involving the protein phosphatase 2a (PP2A), high levels of which were shown to down-regulate autophagy in dependence of methionine availability [31] and (ii) a methionine-independent mechanism, where high acetate levels block autophagy induction [32]. In our MetR setup, the first mechanism does not seem to play a major role since the MET^+^ strain lacking *PPM1* (the methyltransferase of PP2A), did not lead to better chronological survival and deletions in *PPH21* or *PPH22* (catalytic subunits of the PP2A complex) had only small positive effects (Figure S7A). Instead, acetate levels in the media of the MET^+^ strain were about 80% higher compared to those of Δ*met15* and Δ*met2* strains on day 1, reaching comparable (Δ*met2*) or lower levels (Δ*met15*) on day 2 (Figure S7B) of chronological aging. Given the complex regulatory network PP2A is involved in and the metabolic and regulatory implications high acetate levels potentially lead to, contributions to autophagy induction are likely dependent on growth conditions and molecular fine-tuning. However, both pathways potentially influence the TOR pathway or its downstream targets thus supporting our epistasis analysis of MetR and TOR inhibition. Future work will be needed to decipher the specific contributions of these pathways/metabolites under different longevity-mediating regimens.

Altogether, methionine as an ubiquitous factor within cell metabolism may impact aging through several mechanisms that share or are independent from the herein described, for instance, a recent study suggests that methionine regulates homeostasis through modulation of tRNA thiolation and thus translation capacity [33].

Furthermore, we could clearly demonstrate that the proportion of cells displaying an acidic vacuole within a population is significantly enhanced via MetR inflicted either by genetic deletion of *met2* and external methionine availability, or rapamycin treatment of MET^+^. Additionally, we show that this enhancement is strictly dependent on functional autophagy (as shown by an *ATG5* deletion). In line, increasing v-ATPase activity (by overexpression of Vph2p or Vma1p), a process already shown to increase vacuolar acidity [15], is sufficient to increase CLS in a methionine-prototrophic strain. Conclusively, deletion of VPH2 and thus disruption of v-ATPase activity reverses the positive effects of MetR on CLS. Intriguingly, it has been recently demonstrated that an increase in vacuolar pH, specifically during early age, negatively influences the replicative lifespan of yeast [15]. In the same line, a recently published screen for chemical compounds extending CLS in *Schizosaccharomyces pombe* identified, among others, vacuolar acidification as a key process [34]. Additionally, Hughes and Gottschling showed that decreased vacuolar pH positively impacts mitochondrial function [15]. Interestingly, others have determined that autophagy is required to maintain respiration proficiency under caloric restriction conditions in galactose media [29], highlighting a protective role of autophagy, especially for mitochondrial function. In the frame of these observations, the decrease in ROS production during MetR may suggest a mechanistic structure that couples MetR-induced autophagy and vacuolar acidification to mitochondrial function.

In studies relating longevity to autophagy, doubts can be raised on the interpretation of the negative effects of genetic autophagy defects because the deletion of ATG genes may perturb cell survival *per se* [35]. However, we find that in our experimental setup, cells deleted for ATG genes, in fact, display normal growth rates and survival during the first days of chronological aging. Furthermore, we clearly demonstrate that an additional *RAS2* deletion in an autophagy-deficient Δ*met2* strain enhances the mutant's CLS. Nevertheless, the positive effects of a *RAS2* deletion during chronological aging under MetR conditions seem to be limited since longevity is only marginally enhanced in a Δ*met2* strain. This points towards the concept that autophagy is a process that significantly contributes to enhanced longevity upon *RAS2* deletion.

Our work clarifies two further, thus far unexplained issues that are of great importance to researchers working on (yeast) aging: First, we demonstrate that the heterogeneity in CLS of the EUROSCARF wild-type strains BY4741 and BY4742 can be explained by strain-dependent differences in methionine biosynthesis. Second, we show that treatment with rapamycin or deletion of *TOR1* almost only increases the longevity of strains that are methionine-prototrophic. Intriguingly, despite one study that could show enhanced CLS by *tor1* deletion in BY4741 [36], previously published results on extension of CLS via *TOR1* inhibition were performed in strains that are prototrophic for methionine [8], [24], [37]. We can also show that early phases of MetR-mediated autophagy are largely dependent on *TOR1* or impact the same downstream targets. At the same time, there seem to be additional *TOR1*-independent mechanisms of MetR-induced autophagy later on during CLS, which will need to be addressed in future studies. Taken together, we show that autophagy-mediated vacuolar acidification is essential for the anti-aging effects of MetR, one of the rare lifespan-extending scenarios that is conserved across species.

## Materials and Methods

### Yeast strains and media

Experiments were carried out in strains using the EUROSCARF strain collection as basis and are listed in Table S1. In brief: BY4741 (MATa *his3*Δ*1 leu2*Δ*0 met15*Δ*0 ura3*Δ*0*) was used as Δ*met15* strain. MET^+^ was constructed by crossing BY4741 with BY4742 and tetrad selection for being methionine prototroph but otherwise isogenic to BY4741. Accordingly Δ*met2* was generated by crossing BY4741 Δ*met2::kanMX* (EUROSCARF) with BY4742 and tetrad selection for tetra-type (all four spores are methionine auxotroph) and further selection for geneticin resistance (only conferred by Δ*met2::kanMX*). All other deletion strains and chromosomal GFP tagging in these backgrounds were carried out by classical homologous recombination using the pUG and pYM vector systems [38]–[40], and controlled by PCR. Transformation was done using the lithium acetate method [41]. Notably, at least two different clones were tested for any experiment with these newly transformed strains to rule out clonogenic variation of the observed effects. To generate strains with chromosomal mCherry tags at the C-terminus of the vacuolar membrane protein Vph1 plasmid pFA6a3mcherry-natNT2 or pFA6a3mcherry-hphNT1was used for PCR amplification. After transformation and selection, correct integration was tested via PCR and fluorescent microscopy. For overexpression studies of *VPH2*, *VMA1*, and *ATG8* genes were amplified by PCR and inserted into the pESC-HIS vector (Stratagene). Resulting plasmids were verified by sequencing by eurofins/MWG, transformed via the lithium acetate method and subsequently expression was verified by western blot analysis.

All strains were grown on SC medium containing 0.17% yeast nitrogen base (BD Diagnostics; without ammonium sulfate and amino acids), 0.5% (NH_4_)_2_SO_4_, 30 mg/L of all amino acids (aa) (except 80 mg/liter histidine and 200 mg/liter leucine), 30 mg/L adenine, and 320 mg/L uracil with 2% glucose (SCD). All amino acids were purchased from Serva (research grade, ≥98.5%). For experiments with varying methionine or cysteine concentrations, methionine and cysteine were added at given concentrations. For overexpression with the pESC-HIS system strains were grown in the absence of histidine. When needed, glutathione was added at given concentrations at the time of inoculation. Survival plating was done on YPD agar plates (2% peptone, 1% yeast extract, 2% glucose, and 2% agar) and incubated for 2 to 3 days at 28°C.

### Chronological aging, shift aging and overexpression aging

For chronological aging experiments cells were inoculated to 5×10^5^ cells, or alternatively to an OD600 of 0.05, and grown for the indicated time period at 28°C in SC media. If not stated otherwise standard concentration of methionine (30 mg/l) was used throughout the experiments. Shift aging experiments with the Δ*met2* strain were inoculated accordingly grown for 24 hours in excess of methionine (30 mg/l) and subsequently shifted to fresh SC media with indicated amounts of methionine. Of note For overexpression studies strains carrying *VPH2*, *VMA1*, or *ATG8* on a pESC-HIS vector were grown in SCD for 6 hours and subsequently shifted into minimal media containing 0.5% galactose and 1.5% glucose, or 2% galactose (*ATG8*), for induction of expression. At the indicated time points cell survival was determined by clonogenictiy: Cell cultures were counted with a CASY cell counter (Schärfe System) and 500 cells were plated on YPD agar plates. Subsequently colony forming units were counted and values were normalized to survival at day one. Alternatively, cell death was measured via propidium iodide staining and subsequent flow cytometry analysis (BD FACSAria). Representative aging analyses are shown with at least three independent cultures aged at the same time. All aging analyses were performed at least twice in total with similar outcome.

Experiments involving treatment with rapamycin (LC laboratories) were performed as described above. To circumvent growth effects described in other publications rapamycin was added eight hours after inoculation and to lower amounts.

### Tests for cell death markers, pH measurement, oxygen consumption, acidic vacuole staining, and cell cycle analysis

Dihydroethidium (DHE; working concentration: 2.5 µg/ml in PBS; ∼1×10^6^ cells; incubation time 5 to 10 min at RT) staining (ROS production) and Annexin V/propidium iodide costaining (apoptosis/necrosis marker) were performed and quantified by using a fluorescent plate reader (Tecan, GeniusPRO) or by flow cytometry (BD FACSAria) as previously described [42]. 30,000 cells per sample were evaluated using BD FACSDiva software.

Measurement of growth media pH was performed at the indicated time points using a pH-Meter (Metrohm).

Oxygen consumption was determined eight hours after addition of rapamycin using an Oxygraph (Clark-type oxygen electrode connected to an ISO2 recorder; World Precision Instruments) and subsequent data processing with LabChart (ADInstruments).

Quinacrine (Sigma) was used to stain for acidic vacuoles following standard protocols [15]. Briefly, ∼2×10^6^ cells were harvested, washed with YPD containing HEPES buffer (100 mM, pH7.6) and then collected and re-susupended in fresh YPD-HEPES containing 200 µM quinacrine After 10 min incubation at 30°C cells were put on ice and washed three times with ice-cold HEPES buffer containing 2% glucose an finally resuspended in the same buffer. All samples were kept on ice until they were viewed under the microscope within 1 hour since sample taking.

DNA content was measured as described previously [43]. Briefly, ∼1×10^7^ cells were harvested, re-suspended in cold water and fixed with ice cold ethanol. After ∼16 h cells were harvested, re-suspended in sodium-citrate buffer (50 mM, pH7.4) and sonicated. After treatment with RNase and proteinase K, cells were stained overnight with propidium iodide (8 µg/ml), and analyzed by flow cytometry (BD LSRFortessa). 30,000 cells per sample were evaluated using BD FACSDiva software.

### Microscopy

Microscopy of Quincarine stained cells as well as GFP-Atg8p expressing cells was performed with a *Zeiss Axioskop* microscope using a *Zeiss Plan-Neofluar* objective lens with 63× magnification and 1.25 numerical aperture or 40× magnification and 2.0 numerical aperture in oil (using Zeiss Immersol) at room temperature. Fluorescence microscopic sample images were taken with a *Diagnostic Instruments* camera (Model: SPOT 9.0 Monochrome-6), acquired and processed (coloring) using the Metamorph software (version 6.2r4, Universal Imaging Corp.) For creating a statistical analysis 330–600 cells of each GFP-Atg8p expressing strain were evaluated from two independent samples (Figure 3B). For statistical analysis of Quinacrine stained cells, >1000 cells of each strain from 3 to 5 independent samples at each time point were evaluated (Figure 5B and Figure S6B) or >500 cells of each condition from two independent samples (Figure 5C).

### Alkaline phosphatase assay

Autophagy was monitored by alkaline phosphatase (ALP) activity [44]. Strains were transformed with and selected for stable insertion of pTN9 *Hin*dIII fragment (confirmed by PCR). Briefly 1–5×10E7 cells were collected (and kept on ice from that moment on), washed, resuspended in assay buffer (Tris-HCl, 250 mM; pH = 9; 10 mM magnesium phosphate; 10 µM zinc sulfate), disrupted with glass beads, and centrifuged. Protein concentration was determined in the supernatant via a Bradford assay (BioRad) following standard protocols and subsequently 1 µg of total protein extract was subjected to the ALP assay. Extracts were incubated with α-naphtyl phosphate (55 mM) for 20 min at 30°C and stopped with 2 M glycine-NaOH (pH = 11). To correct for intrinsic (background) ALP activity, the corresponding strains without pTN9 were simultaneously processed and ALP activity was subtracted. Alternatively, strains were transformed with and selected for pCC5 (a plasmid carrying the cytoplasmic *PHO8Δ60* [45]). Relative fluorescence units (RFU) were determined by using a fluorescence reader (Tecan, GeniusPRO) and applying the same manual gain throughout a series of measurements belonging together. For each transformed strain, two clones were tested.

### Immunoblotting

Preparation of cell extracts and immunoblotting were performed as described [46]. Blots were probed with monoclonal mouse anti-GFP antibody (Roche, Cat.No:11814460001), rabbit polyclonal antibodies against glyceraldehyde-3-phosphate dehydrogenase (gift from Günther Daum) and the respective peroxidase-conjugated affinity-purified secondary antibody (Anti-Mouse IgG-Peroxidase antibody A9044 and Anti-Rabbit IgG-Peroxidase antibody A0545, Sigma). For detection the ECL system was used (Amersham).

### Statistical analyses

Error bars (± SEM) are shown for independent experiments/samples. In cases when experiments were performed in parallel, a common overnight culture (ONC) for each strain was used. The number of independent data points (*n*) is indicated in the figure legends of the corresponding graphs. Significances were calculated using students t-test (one-tailed, unpaired). For aging experiments, a two-factor ANOVA with strain and time as independent factors was applied and corrected by the Bonferroni post hoc test. Significances: *p<0.05, **p<0.01, ***p<0.001.

## Supporting Information

Figure S1 (A) Cell count during chronological aging experiments of methionine prototroph (MET^+^), semi-auxotroph (Δ*met15*) and auxotroph (Δ*met2*) isogenic yeast strains in SCD media supplemented with all amino acids (aa) (n = 4). (B) Cell cycle staining of MET^+^, Δ*met15* and Δ*met2* strains during day 1 and 2 of a CLS experiment. (C) Media pH of a chronological aging experiment of MET^+^, Δ*met15*, and Δ*met2* strain, respectively, at indicated time points (n = 4). (D) Chronological aging of MET^+^ strain, in SCD media supplemented with all aa except for methionine which was added at given concentrations. Cell survival of 500 cells plated at given time points, normalized to cell survival on day one and cell count thereof (E) (n = 4). (F) Chronological aging of MET^+^ strain, in SCD media supplemented with all aa except for methionine which was added at given concentrations. Cell survival of 500 cells plated at given time points, normalized to cell survival on day one and cell count thereof (G) (n = 4). (H) Chronological aging of MET^+^ strain, in SCD media supplemented with all aa except for cysteine which was added at given concentrations. Cell survival of 500 cells plated at given time points, normalized to cell survival on day one (n = 4). (I) Cell count of *MET2* deletion strain (Δ*met2*) during chronological aging in SCD media supplemented with all aa except for methionine which was added at given concentrations (n = 4).
(TIF)

Figure S2 MET^+^, Δ*met15* and Δ*met2* strains aged in SCD media (supplemented with all aa) were stained for necrotic and apoptotic markers at indicated time points. (A) Externalization of phosphatidyl-serine was determined by AnnexinV/PI co-staining and analyzed by flow cytometry (BD, FACS-Aria) (n = 4). (B) Reactive oxygen species (ROS) were determined by conversion of DHE to Ethidium (Eth) and analyzed with a fluorescent plate reader (Tecan, Genios Pro) (n = 10).
(TIF)

Figure S3 (A) Chronological aging of the MET^+^ strain overexpressing Atg8p. Cell death was measured via propidium iodide staining of cells that have lost integrity and subsequent flow cytometry analysis (BD LSRFortessa) (n = 6). ALP assays of chronological aging of Δ*met15* (B) and Δ*met2* strain (C), in SCD media supplemented with all aa except for methionine which was added at given concentrations. Analyses were performed on a fluorescent plate reader (Tecan, Genios Pro) (n = 2 to 4).
(TIF)

Figure S4 (A) Chronological aging of *MET2* deletion strains carrying single gene ATG deletions (Δ*atg7*, and Δ*atg8*, respectively) compared to MET^+^ strain in SCD media supplemented with all aa. Cell survival of 500 cells plated at given time points, normalized to cell survival on day one (n = 8). (B) Chronological aging of *MET15* deletion strains carrying single gene ATG deletions (Δ*atg5*, Δ*atg7*, and Δ*atg8*, respectively) (n = 4 to 6). (C) Chronological aging of *MET2* deletion strains carrying additional gene deletions (Δ*atg5* and/or Δ*ras2*) compared to MET^+^ (n = 4 to 6, respectively; Note: data of strain Δ*met2* Δ*atg5* was added from a separate experiment series). (D) Chronological aging of the MET^+^ strain deleted for *ATG5*, *ATG7* or *ATG8*. Cell survival of 500 cells plated at given time points, normalized to cell survival on day one (n = 4 to 6). (E and F) Chronological aging experiment of MET^+^ strain treated with indicated amounts of rapamycin (Rap). (E) Cell count measured with a CASY cell counter at given time points (n = 3). (F) O_2_ consumption in logarithmic growth phase, eight hours after addition of indicated amounts of rapamycin (n = 8). (G) Chronological aging of the MET^+^ strain deleted for *TOR1* and *ATG7* or *ATG7* alone. Cell survival of 500 cells plated at given time points, normalized to cell survival on day one (n = 4 to 6). (H) Chronological aging of the MET^+^ strain deleted for *TOR1* and *ATG8* or *ATG8* alone. Cell survival of 500 cells plated at given time points, normalized to cell survival on day one (n = 4 to 6).
(TIF)

Figure S5 (A and B) Chronological aging experiment of Δ*met2* or Δ*met15* strains treated with glutathione. Cell survival of 500 cells plated at given time points, normalized to cell survival on day one (n = 4). (C) ALP assays of *MET2*/*TOR1* deletion strain, grown to stationary phase in SCD (supplemented with all aa) and shifted to SCD media with indicated methionine concentrations. Analyses were performed on a fluorescent plate reader (Tecan, Genios Pro) (n = 4), and ALP activity was normalized to values of samples done in SCD with 30 mg/l methionine. (D and E) Cell counts during chronological aging experiments of Δ*met2* or Δ*met15* strains treated with indicated amounts of rapamycin (Rap), measured with a CASY cell counter at given time points (n = 3).
(TIF)

Figure S6 (A) Fluorescent microscopy of acidic vacuoles during chronological aging of MET+, Δ*met2*, and Δ*met2*/Δ*atg5* strains carrying chromosomally *VPH1*-mCherry to visualize the vacuolar membrane, by means of quinacrine accumulation. (B) Statistical analysis of fluorescent microscopy of acidic vacuoles by means of quinacrine accumulation during a chronological aging of MET^+^ strain treated with indicated amounts of rapamycin (Rap). Note: Rapamycin strongly increased small acidic compartments/vesicles. (>1000 cells of each strain from 3 to 5 independent samples at each time point were evaluated). (C) Chronological aging of Δ*met2* and Δ*met2*/Δ*vph2* strains compared to the MET^+^ strain in SCD media supplemented with all aa. Cell survival of 500 cells plated at given time points, normalized to cell survival on day one (n = 4) Note: MET+ strain deleted for *vph2* did deliver mutants with an instable aging phenotype. (D) Fluorescent microscopy of acidic vacuoles on day 1 and 3 of chronological aging in a Δ*met2*/Δ*vph2* strain, by means of quinacrine accumulation. (E) Chronological aging of *MET2* deletion strains overexpressing Vma1p or Vph2p. Cell death was measured via propidium iodide staining of cells that have lost integrity and subsequent flow cytometry analysis (BD LSRFortessa) (n = 4 to 8).
(TIF)

Figure S7 (A) Chronological aging of methionine prototroph (MET^+^) strain and isogenic strains carrying single gene deletions (Δ*ppm1*, Δ*pph21* or Δ*pph22*) in SCD media supplemented with all amino acids (aa). Cell survival was estimated as colony formation of 500 cells plated at given time points, normalized to cell survival on day one (n = 6). (B) Media acetate levels of methionine prototroph (MET^+^), semi-auxotroph (Δ*met15*) and auxotroph (Δ*met2*) on days one and two during chronological aging experiments (n = 8).
(TIF)

Table S1 *S. cerevisiae* strains used in this study.
(DOCX)
