# Supplementary figures and images for: Lifespan Extension by Methionine Restriction Requires Autophagy-Dependent Vacuolar Acidification
Source: PLoS Genet. 2014 May 1;10(5):e1004347. doi: 10.1371/journal.pgen.1004347 (PMC4006742; doi:10.1371/journal.pgen.1004347)

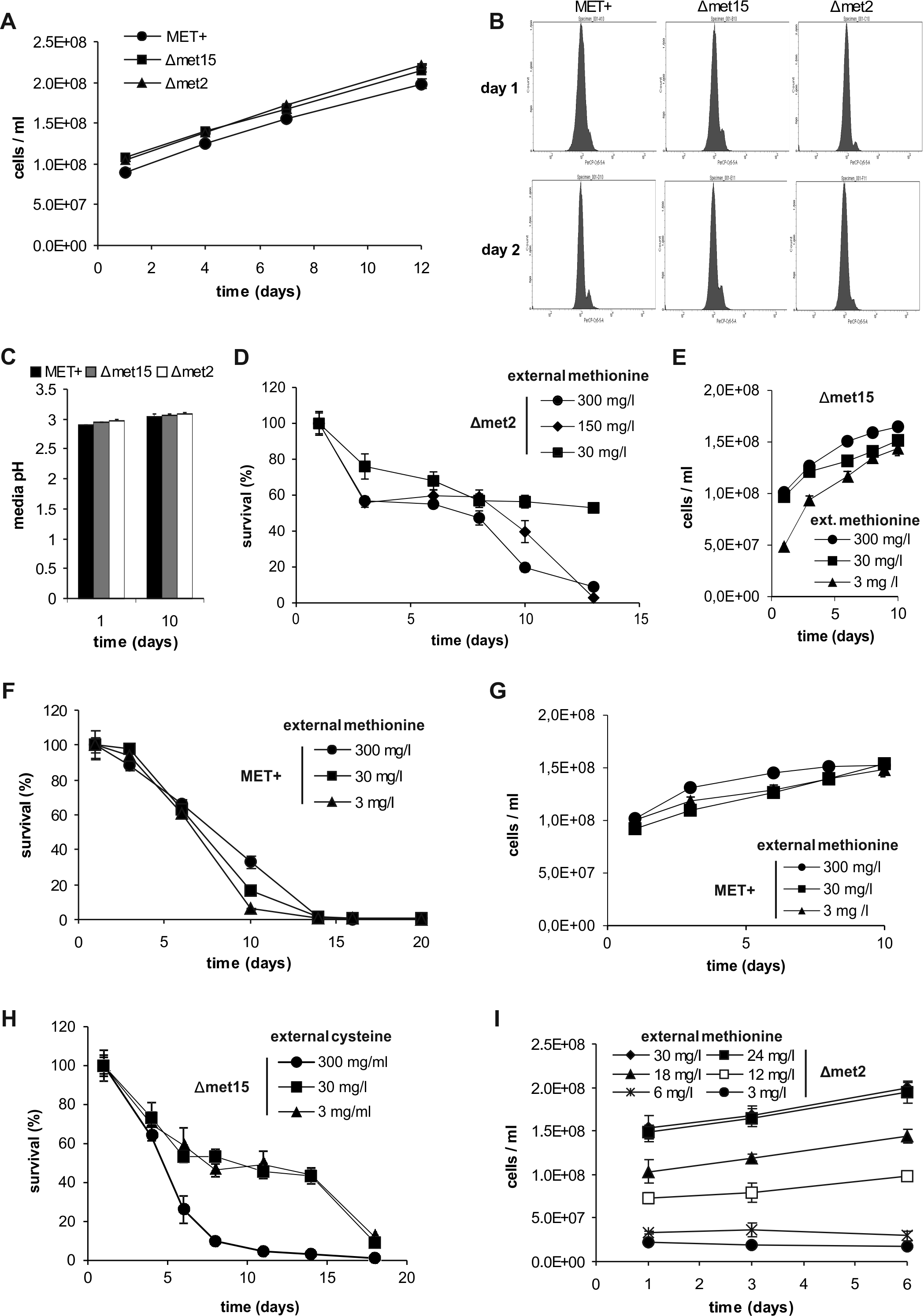

Supplement: Figure S1 — (A) Cell count during chronological aging experiments of methionine prototroph (MET+), semi-auxotroph (Δmet15) and auxotroph (Δmet2) isogenic yeast strains in SCD media supplemented with all amino acids (aa) (n = 4). (B) Cell cycle staining of MET+, Δmet15 and Δmet2 strains during day 1 and 2 of a CLS experiment. (C) Media pH of a chronological aging experiment of MET+, Δmet15, and Δmet2 strain, respectively, at indicated time points (n = 4). (D) Chronological aging of MET+ strain, in SCD media supplemented with all aa except for methionine which was added at given concentrations. Cell survival of 500 cells plated at given time points, normalized to cell survival on day one and cell count thereof (E) (n = 4). (F) Chronological aging of MET+ strain, in SCD media supplemented with all aa except for methionine which was added at given concentrations. Cell survival of 500 cells plated at given time points, normalized to cell survival on day one and cell count thereof (G) (n = 4). (H) Chronological aging of MET+ strain, in SCD media supplemented with all aa except for cysteine which was added at given concentrations. Cell survival of 500 cells plated at given time points, normalized to cell survival on day one (n = 4). (I) Cell count of MET2 deletion strain (Δmet2) during chronological aging in SCD media supplemented with all aa except for methionine which was added at given concentrations (n = 4). (TIF) [file pgen.1004347.s001.tif]

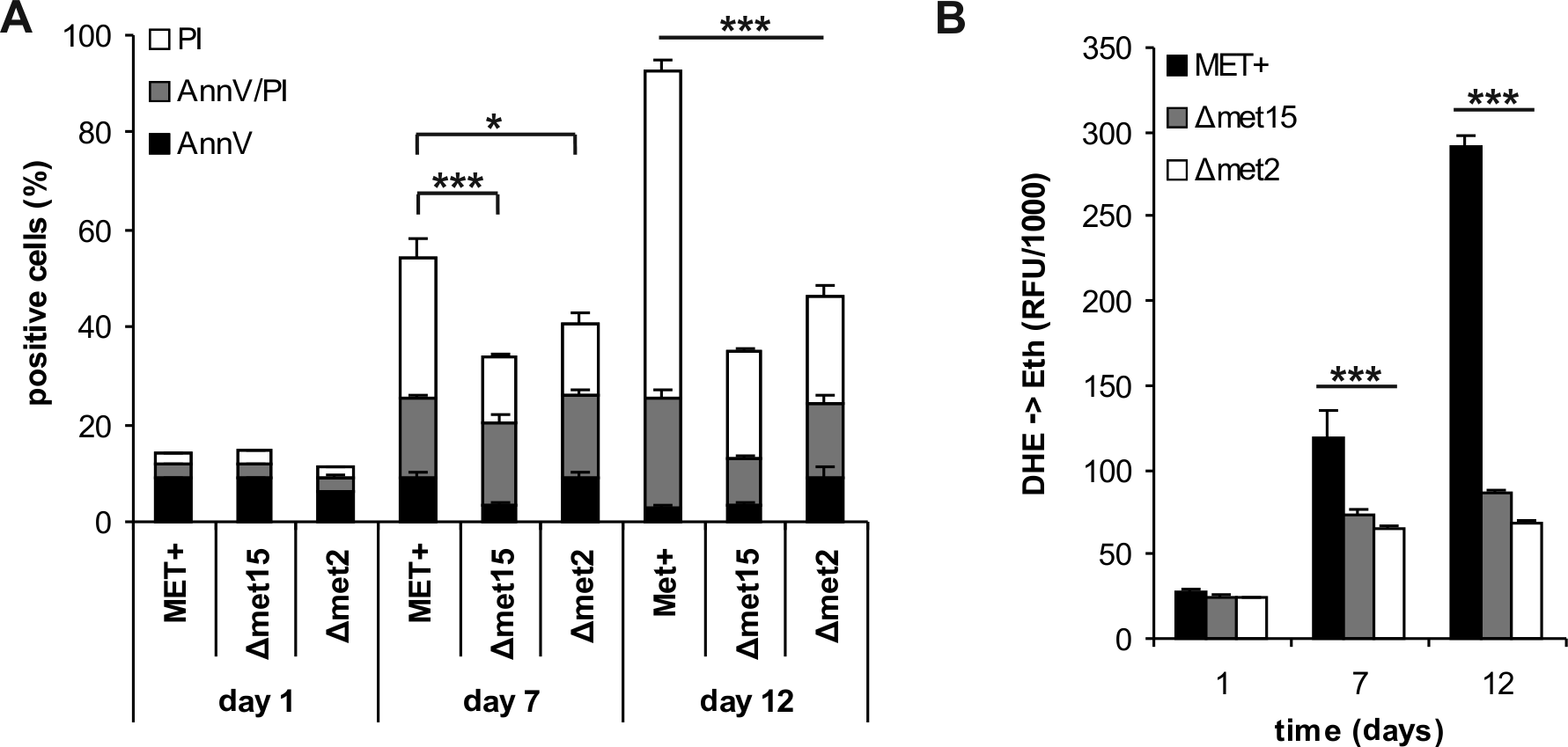

Supplement: Figure S2 — MET+, Δmet15 and Δmet2 strains aged in SCD media (supplemented with all aa) were stained for necrotic and apoptotic markers at indicated time points. (A) Externalization of phosphatidyl-serine was determined by AnnexinV/PI co-staining and analyzed by flow cytometry (BD, FACS-Aria) (n = 4). (B) Reactive oxygen species (ROS) were determined by conversion of DHE to Ethidium (Eth) and analyzed with a fluorescent plate reader (Tecan, Genios Pro) (n = 10). (TIF) [file pgen.1004347.s002.tif]

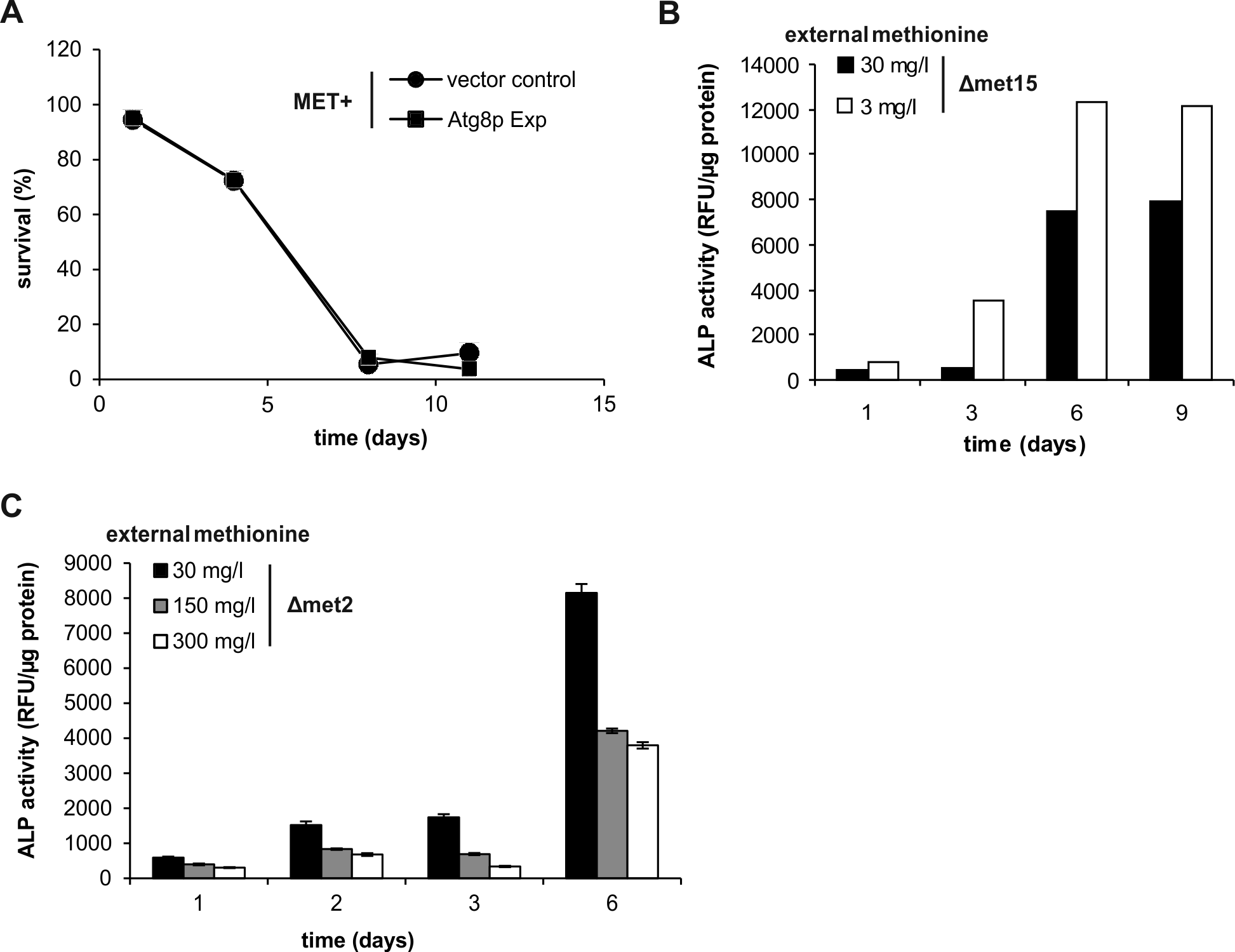

Supplement: Figure S3 — (A) Chronological aging of the MET+ strain overexpressing Atg8p. Cell death was measured via propidium iodide staining of cells that have lost integrity and subsequent flow cytometry analysis (BD LSRFortessa) (n = 6). ALP assays of chronological aging of Δmet15 (B) and Δmet2 strain (C), in SCD media supplemented with all aa except for methionine which was added at given concentrations. Analyses were performed on a fluorescent plate reader (Tecan, Genios Pro) (n = 2 to 4). (TIF) [file pgen.1004347.s003.tif]

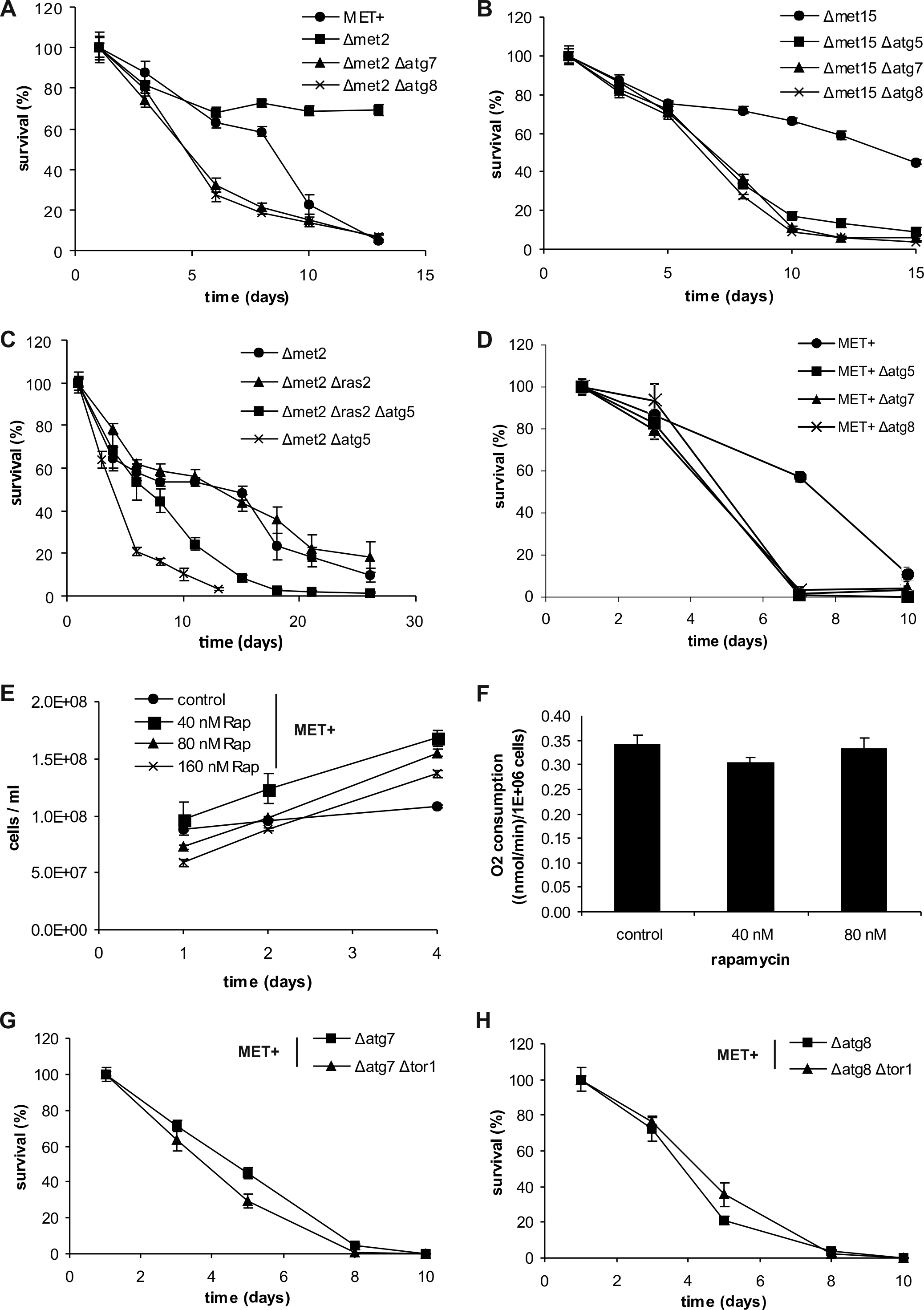

Supplement: Figure S4 — (A) Chronological aging of MET2 deletion strains carrying single gene ATG deletions (Δatg7, and Δatg8, respectively) compared to MET+ strain in SCD media supplemented with all aa. Cell survival of 500 cells plated at given time points, normalized to cell survival on day one (n = 8). (B) Chronological aging of MET15 deletion strains carrying single gene ATG deletions (Δatg5, Δatg7, and Δatg8, respectively) (n = 4 to 6). (C) Chronological aging of MET2 deletion strains carrying additional gene deletions (Δatg5 and/or Δras2) compared to MET+ (n = 4 to 6, respectively; Note: data of strain Δmet2 Δatg5 was added from a separate experiment series). (D) Chronological aging of the MET+ strain deleted for ATG5, ATG7 or ATG8. Cell survival of 500 cells plated at given time points, normalized to cell survival on day one (n = 4 to 6). (E and F) Chronological aging experiment of MET+ strain treated with indicated amounts of rapamycin (Rap). (E) Cell count measured with a CASY cell counter at given time points (n = 3). (F) O2 consumption in logarithmic growth phase, eight hours after addition of indicated amounts of rapamycin (n = 8). (G) Chronological aging of the MET+ strain deleted for TOR1 and ATG7 or ATG7 alone. Cell survival of 500 cells plated at given time points, normalized to cell survival on day one (n = 4 to 6). (H) Chronological aging of the MET+ strain deleted for TOR1 and ATG8 or ATG8 alone. Cell survival of 500 cells plated at given time points, normalized to cell survival on day one (n = 4 to 6). (TIF) [file pgen.1004347.s004.tif]

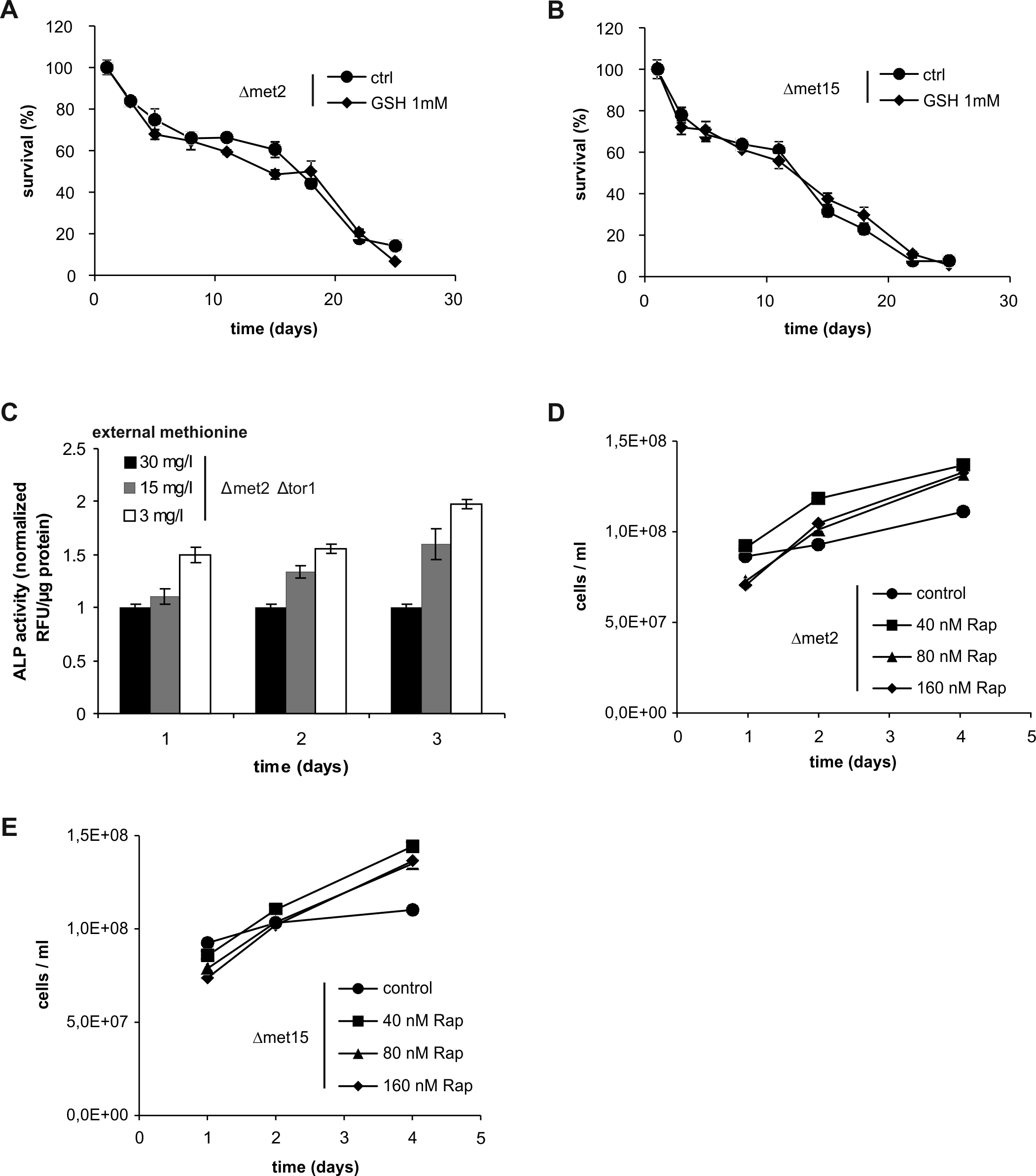

Supplement: Figure S5 — (A and B) Chronological aging experiment of Δmet2 or Δmet15 strains treated with glutathione. Cell survival of 500 cells plated at given time points, normalized to cell survival on day one (n = 4). (C) ALP assays of MET2/TOR1 deletion strain, grown to stationary phase in SCD (supplemented with all aa) and shifted to SCD media with indicated methionine concentrations. Analyses were performed on a fluorescent plate reader (Tecan, Genios Pro) (n = 4), and ALP activity was normalized to values of samples done in SCD with 30 mg/l methionine. (D and E) Cell counts during chronological aging experiments of Δmet2 or Δmet15 strains treated with indicated amounts of rapamycin (Rap), measured with a CASY cell counter at given time points (n = 3). (TIF) [file pgen.1004347.s005.tif]

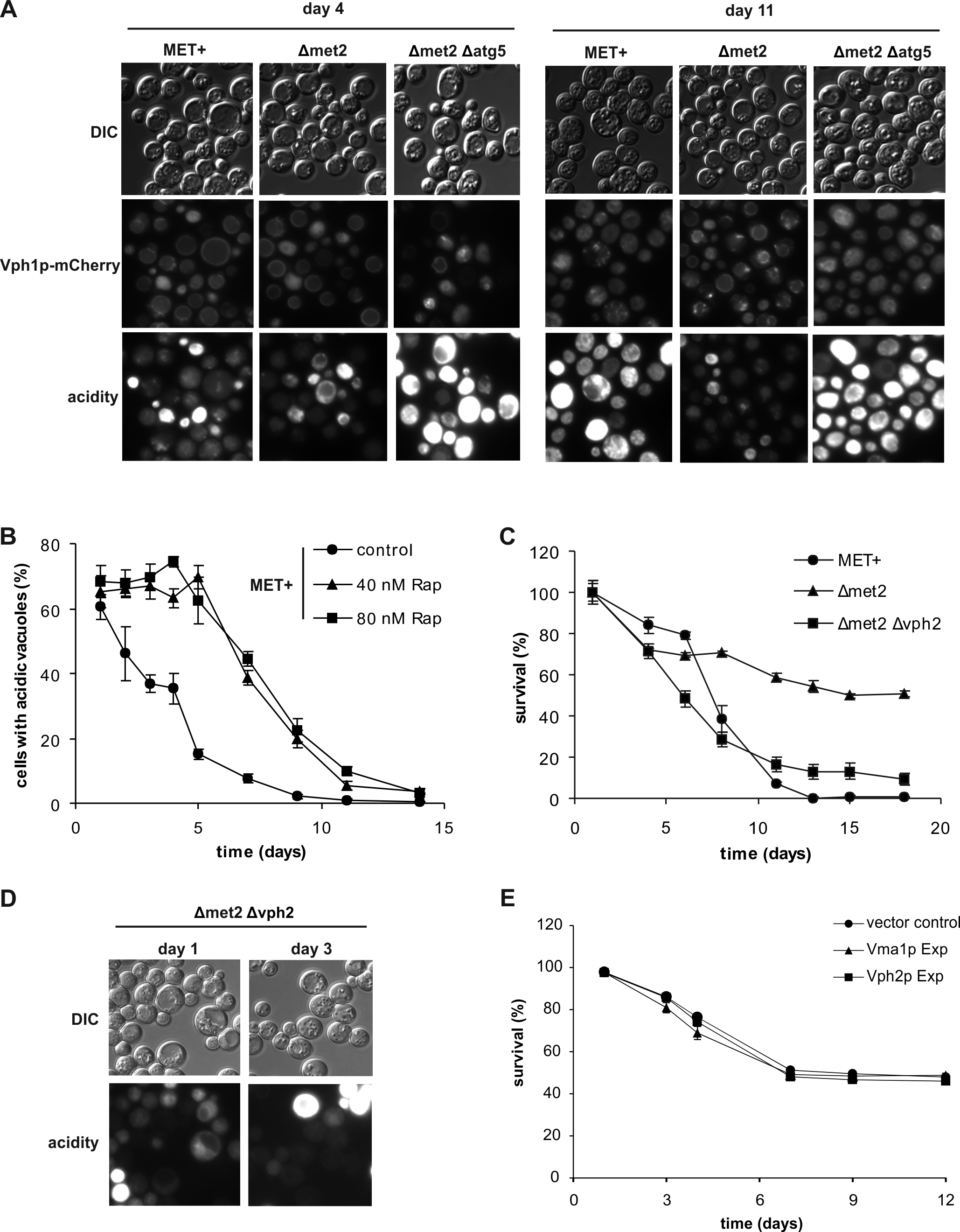

Supplement: Figure S6 — (A) Fluorescent microscopy of acidic vacuoles during chronological aging of MET+, Δmet2, and Δmet2/Δatg5 strains carrying chromosomally VPH1-mCherry to visualize the vacuolar membrane, by means of quinacrine accumulation. (B) Statistical analysis of fluorescent microscopy of acidic vacuoles by means of quinacrine accumulation during a chronological aging of MET+ strain treated with indicated amounts of rapamycin (Rap). Note: Rapamycin strongly increased small acidic compartments/vesicles. (>1000 cells of each strain from 3 to 5 independent samples at each time point were evaluated). (C) Chronological aging of Δmet2 and Δmet2/Δvph2 strains compared to the MET+ strain in SCD media supplemented with all aa. Cell survival of 500 cells plated at given time points, normalized to cell survival on day one (n = 4) Note: MET+ strain deleted for vph2 did deliver mutants with an instable aging phenotype. (D) Fluorescent microscopy of acidic vacuoles on day 1 and 3 of chronological aging in a Δmet2/Δvph2 strain, by means of quinacrine accumulation. (E) Chronological aging of MET2 deletion strains overexpressing Vma1p or Vph2p. Cell death was measured via propidium iodide staining of cells that have lost integrity and subsequent flow cytometry analysis (BD LSRFortessa) (n = 4 to 8). (TIF) [file pgen.1004347.s006.tif]

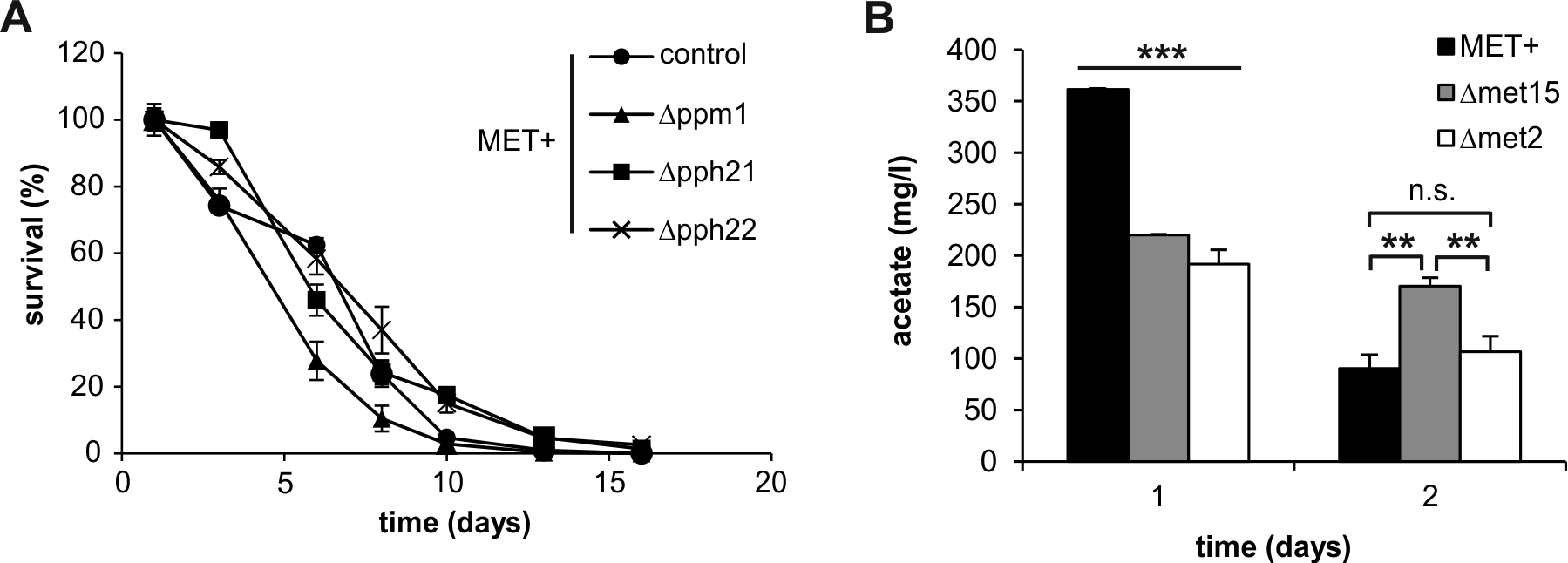

Supplement: Figure S7 — (A) Chronological aging of methionine prototroph (MET+) strain and isogenic strains carrying single gene deletions (Δppm1, Δpph21 or Δpph22) in SCD media supplemented with all amino acids (aa). Cell survival was estimated as colony formation of 500 cells plated at given time points, normalized to cell survival on day one (n = 6). (B) Media acetate levels of methionine prototroph (MET+), semi-auxotroph (Δmet15) and auxotroph (Δmet2) on days one and two during chronological aging experiments (n = 8). (TIF) [file pgen.1004347.s007.tif]
