# Supplementary material for: Lifespan Extension by Methionine Restriction Requires Autophagy-Dependent Vacuolar Acidification
Source: PLoS Genet. 2014 May 1;10(5):e1004347. doi: 10.1371/journal.pgen.1004347 (PMC4006742; doi:10.1371/journal.pgen.1004347)
Supplement: Table S1 — S. cerevisiae strains used in this study. (DOCX) [file pgen.1004347.s008.docx]

**Table S1. *S. cerevisiae* strains used in this study.**

| **Strain** | **Genotype** | **Source** |
| --- | --- | --- |
| Δ*met15 =* BY4741 ^A^ | *MATa his3Δ1 leu2Δ0 met15Δ0 ura3Δ0* | Euroscarf |
| BY4742 | *MATα his3Δ1 leu2Δ0 lys2Δ0 ura3Δ0* | Euroscarf |
| BY4742 Δ*met15* | *MATα his3Δ1 leu2Δ0 lys2Δ0 ura3Δ0 MET15::kanMX* | Euroscarf |
| Δ*met2* | *MATa his3Δ1 leu2Δ0 ura3Δ0 MET2::kanMX* | This study |
| MET^+^ | *MATa his3Δ1 leu2Δ0 ura3Δ0* | This study |
| Δ*met15 GFP-ATG8* | *MATa his3Δ1 leu2Δ0 met15Δ0 ura3Δ0 natNT2:Met^P^-GFP:ATG8* ^B^ | This study |
| Δ*met15* Δ*atg5* | *MATa his3Δ1 leu2Δ0 met15Δ0 ura3Δ0 ATG5::HIS3* | This study |
| Δ*met15* Δ*atg7* | *MATa his3Δ1 leu2Δ0 met15Δ0 ura3Δ0 ATG7::HIS3* | This study |
| Δ*met15* Δ*atg8* | *MATa his3Δ1 leu2Δ0 met15Δ0 ura3Δ0 ATG8::HIS3* | This study |
| Δ*met15* Δ*tor1* | *MATa his3Δ1 leu2Δ0 met15Δ0 ura3Δ0 TOR1::HIS3* | This study |
| Δ*met2 GFP-ATG8* | *MATa his3Δ1 leu2Δ0 ura3Δ0 MET2::kanMX natNT2: Met^P^-GFP:ATG8*^B^ | This study |
| Δ*met2* Δ*atg5* | *MATa his3Δ1 leu2Δ0 ura3Δ0 MET2::kanMX ATG5::HIS3* | This study |
| Δ*met2* Δ*atg7* | *MATa his3Δ1 leu2Δ0 ura3Δ0 MET2::kanMX ATG7::HIS3* | This study |
| Δ*met2* Δ*atg8* | *MATa his3Δ1 leu2Δ0 ura3Δ0 MET2::kanMX ATG9::HIS3* | This study |
| Δ*met2* Δ*tor1* | *MATa his3Δ1 leu2Δ0 ura3Δ0 MET2::kanMX TOR1::HIS3* | This study |
| Δ*met2* Δ*ras2* | *MATa his3Δ1 leu2Δ0 ura3Δ0 MET2::kanMX RAS2::HIS3* | This study |
| Δ*met2* Δ*atg5*Δ*ras2* | *MATa his3Δ1 leu2Δ0 ura3Δ0 MET2::kanMX ATG5::URA3 RAS2::HIS3* | This study |
| Δ*met2* Δ*vph2* | *MATa his3Δ1 leu2Δ0 ura3Δ0 MET2::kanMX VPH2::HIS3* | This study |
| Δ*met2* Vector control | *MATa his3Δ1 leu2Δ0 ura3Δ0* *MET2::kanMX* [pESC] | This study |
| Δ*met2* Vph2p Exp | *MATa his3Δ1 leu2Δ0 ura3Δ0* *MET2::kanMX* [pESC-*VMA1*] | This study |
| Δ*met2* Vma1p Exp | *MATa his3Δ1 leu2Δ0 ura3Δ0* *MET2::kanMX* [pESC-*VPH2*] | This study |
| MET^+^ *GFP-ATG8* | *MATa his3Δ1 leu2Δ0 ura3Δ0 natNT2:Met^P^-GFP:ATG8* ^B^ | This study |
| MET^+^ Δ*atg5* | *MATa his3Δ1 leu2Δ0 ura3Δ0 ATG5::HIS3* | This study |
| MET^+^ Δ*atg7* | *MATa his3Δ1 leu2Δ0 ura3Δ0 ATG7::HIS3* | This study |
| MET^+^ Δ*atg8* | *MATa his3Δ1 leu2Δ0 ura3Δ0 ATG8::HIS3* | This study |
| MET^+^ Δ*tor1* | *MATa his3Δ1 leu2Δ0 ura3Δ0 TOR1::HIS3* | This study |
| MET^+^ Δ*tor1* Δ*atg5* | *MATa his3Δ1 leu2Δ0 ura3Δ0 ATG5::URA3 TOR1::kanMX* | This study |
| MET^+^ Δ*tor1* Δ*atg7* | *MATa his3Δ1 leu2Δ0 ura3Δ0 ATG7::HIS3 TOR1::URA3* | This study |
| MET^+^ Δ*tor1* Δ*atg8* | *MATa his3Δ1 leu2Δ0 ura3Δ0 ATG8::HIS3 TOR1::URA3* | This study |
| MET^+^ *VPH1mCherry* | *MATa his3Δ1 leu2Δ0 ura3Δ0 VPH1:mCherry:hphNT1* ^C^ | This study |
| MET^+^ Δ*ppm1* | *MATa his3Δ1 leu2Δ0 ura3Δ0 PPM1::HIS3* | This study |
| MET^+^ Vector control | *MATa his3Δ1 leu2Δ0 ura3Δ0* [pESC] | This study |
| MET^+^ Vma1p Exp | *MATa his3Δ1 leu2Δ0 ura3Δ0* [pESC-*VMA1*] | This study |
| MET^+^ Vph2p Exp | *MATa his3Δ1 leu2Δ0 ura3Δ0* [pESC-*VPH2*] | This study |
| MET^+^ Atg8p Exp | *MATa his3Δ1 leu2Δ0 ura3Δ0* [pESC-*ATG8*] | This study |

^A^ Standard name for *MET15* is *MET17*

^B^ chromosomal N-terminal GFP-tag, generated by the use of pYM-N37 [36].

^C^ chromosomal C-terminal mCherry-tag, generated by the use of pFA6a3mcherry-natNT2 or pFA6a3mcherry-hphNT1 (both a gift from Elmar Schiebel).
